# Unsupervised deep representation learning enables phenotype discovery for genetic association studies of brain imaging

**DOI:** 10.1038/s42003-024-06096-7

**Published:** 2024-04-05

**Authors:** Khush Patel, Ziqian Xie, Hao Yuan, Sheikh Muhammad Saiful Islam, Yaochen Xie, Wei He, Wanheng Zhang, Assaf Gottlieb, Han Chen, Luca Giancardo, Alexander Knaack, Evan Fletcher, Myriam Fornage, Shuiwang Ji, Degui Zhi

**Affiliations:** 1 McWilliams School of Biomedical Informatics, University of Texas Health Science Center, Houston, TX 77030 USA; 2 Department of Computer Science and Engineering, Texas A&M University, College Station, TX 77843 USA; 3 School of Public Health, University of Texas Health Science Center, Houston, TX 77030 USA; 4 Department of Neurology and Imaging of Dementia and Aging (IDeA) Laboratory, University of California at Davis, Davis, CA 95618 USA; 5 McGovern Medical School, University of Texas Health Science Center, Houston, TX 77030 USA

**Keywords:** Computational biology and bioinformatics, Genetic association study

## Abstract

Understanding the genetic architecture of brain structure is challenging, partly due to difficulties in designing robust, non-biased descriptors of brain morphology. Until recently, brain measures for genome-wide association studies (GWAS) consisted of traditionally expert-defined or software-derived image-derived phenotypes (IDPs) that are often based on theoretical preconceptions or computed from limited amounts of data. Here, we present an approach to derive brain imaging phenotypes using unsupervised deep representation learning. We train a 3-D convolutional autoencoder model with reconstruction loss on 6130 UK Biobank (UKBB) participants’ T1 or T2-FLAIR (T2) brain MRIs to create a 128-dimensional representation known as Unsupervised Deep learning derived Imaging Phenotypes (UDIPs). GWAS of these UDIPs in held-out UKBB subjects (n = 22,880 discovery and n = 12,359/11,265 replication cohorts for T1/T2) identified 9457 significant SNPs organized into 97 independent genetic loci of which 60 loci were replicated. Twenty-six loci were not reported in earlier T1 and T2 IDP-based UK Biobank GWAS. We developed a perturbation-based decoder interpretation approach to show that these loci are associated with UDIPs mapped to multiple relevant brain regions. Our results established unsupervised deep learning can derive robust, unbiased, heritable, and interpretable brain imaging phenotypes.

## Abstract (web summary)

A study utilizing unsupervised deep learning to generate interpretable brain imaging phenotypes from brain T1 and T2-FLAIR MRI identified 97 genetic loci enhancing understanding of brain structure genetics.

## Introduction

Structural magnetic resonance imaging (MRI) modalities such as T1 and T2-FLAIR (T2) scans enable the study of brain anatomy and pathology in high resolution. With the availability of large cohorts with both brain MRI and genetic information^1–5^, genome-wide association studies (GWAS) of the brain structures have shed light on the genetic factors underlying the variations in brain morphology and can potentially aid the understanding of etiopathology of neuropsychiatric disorders. Still, one of the main methodological challenges for brain imaging GWAS is to derive comprehensive, heritable, and interpretable representations of the brain from complex 3D brain MRIs. Most existing GWAS studies^6–10^ use phenotype values capturing volumes of brain regions, cortical surface area and cortex thickness estimated by classical software such as FSL^11^, FreeSurfer^12^ and SPM^13^. One of the most extensive efforts of this type was the genetic studies of the UK Biobank (UKBB) brain imaging data^14–17^. In particular, an extensive set of 3144 brain imaging-derived phenotypes (IDPs), directly measurable features derived from brain images by algorithmic processing, including 1437 descriptors of brain structure derived from T1 and T2 images, from the 33,224 UKBB participants was studied and 692 clusters of association between genetic variants and IDPs were identified^15^. Also, a GWAS of FreeSurfer-derived vertex-based measures using UKBB data identified 780 loci for cortical thickness and surface area^16^.

However, traditional approaches for deriving phenotypes from brain MRI have limitations. Brain-derived IDPs are always subject to ambiguity and uncertainty due to many factors. For example, segmentation of brain into regions of interest (ROIs) was often a prerequisite for downstream processing. However, even widely used standard region segmentation software still has biases and inconsistencies^18,19^. Also, the brain segmentation algorithms based on image registration may be affected significantly by artifacts and pathology^20–22^.

In addition to issues arising from traditional approaches to segmenting brain IDPs, recent commentaries have pointed out inherent limitations from modeling outcomes associated with single brain measures from pre-selected lists^23^. The essence of this critique is that portions of multiple IDPs may be associated with an outcome of interest (e.g., cognitive performance or genetic association), crossing traditional ROI boundaries to recruit subsets of multiple regions while not using the entirety of any region. Modeling brain-outcome associations with single whole IDPs thus loses both anatomical specificity (by forcibly incorporating more of a single IDP than is really associated to outcome) and sensitivity (failing to incorporate portions of other IDPs that are also associated)^23^. This is true a fortiori in GWAS, due to the pleiotropy or distributed influence of a genetic locus (SNP) on multiple brain regions and across different imaging modalities^24^.

In response, some multivariate statistical approaches have modeled SNP associations with multiple brain measures, obtaining increased sensitivity and enhanced loci discovery. However, these approaches still require input of precomputed brain measures in some form. This necessitates a priori selections of measures that are expected to be relevant, with the risk of overlooking others. For example, a multivariate association study of cortical vertices^16^ captures some cortical features but omits subcortical features that could also discover important brain-genome relations.

In the general MRI imaging domain, deep learning (DL) methods, especially convolutional neural networks (CNNs), have demonstrated their power to learn useful features for predicting brain-related phenotypes^25^. However, there has been minimal success in using deep learning to generate brain imaging phenotypes for GWAS. One of the misconceptions is that extensive labeling is needed to train a DL model for phenotyping MRIs.

Here, we propose unsupervised learning for deriving phenotypes from brain MRIs.

Our deep learning approach has the potential to address sensitivity and specificity issues without requiring a precomputed set of brain measures. Specifically, we trained 3D convolutional autoencoders with reconstruction loss and used the bottleneck layer as a vector representation for the input MRI. This unsupervised approach computes a set of latent brain measures that implicitly combine features of the whole brain image to best encode individual brains in large training sets. The only criterion in the training is the ability to reproduce an image from its encoding. Since the encoding is a data reduction, there will be loss, but this is determined by the architecture of the neural net rather than an a priori judgment as to which brain features are relevant. Therefore, our approach has the potential to go beyond a priori measurements to generate measures with improved power for genetic discovery.

In this study, we trained our model on UKBB’s T1 and T2 MRIs. The extracted features in the bottleneck layer were then used as Unsupervised Deep learning derived Imaging Phenotypes (UDIPs) for GWAS. Using the decoder as the generator, a perturbation-based decoder interpretation (PerDI) approach was developed to map the UDIPs to brain regions. These visual associations were corroborated by results from prior research. Our results suggest that this label-free approach can be used to derive interpretable and heritable phenotypes, empowering the discovery of the genetic architecture of the brain.

## Results

### Overview

**Fig. 1:**
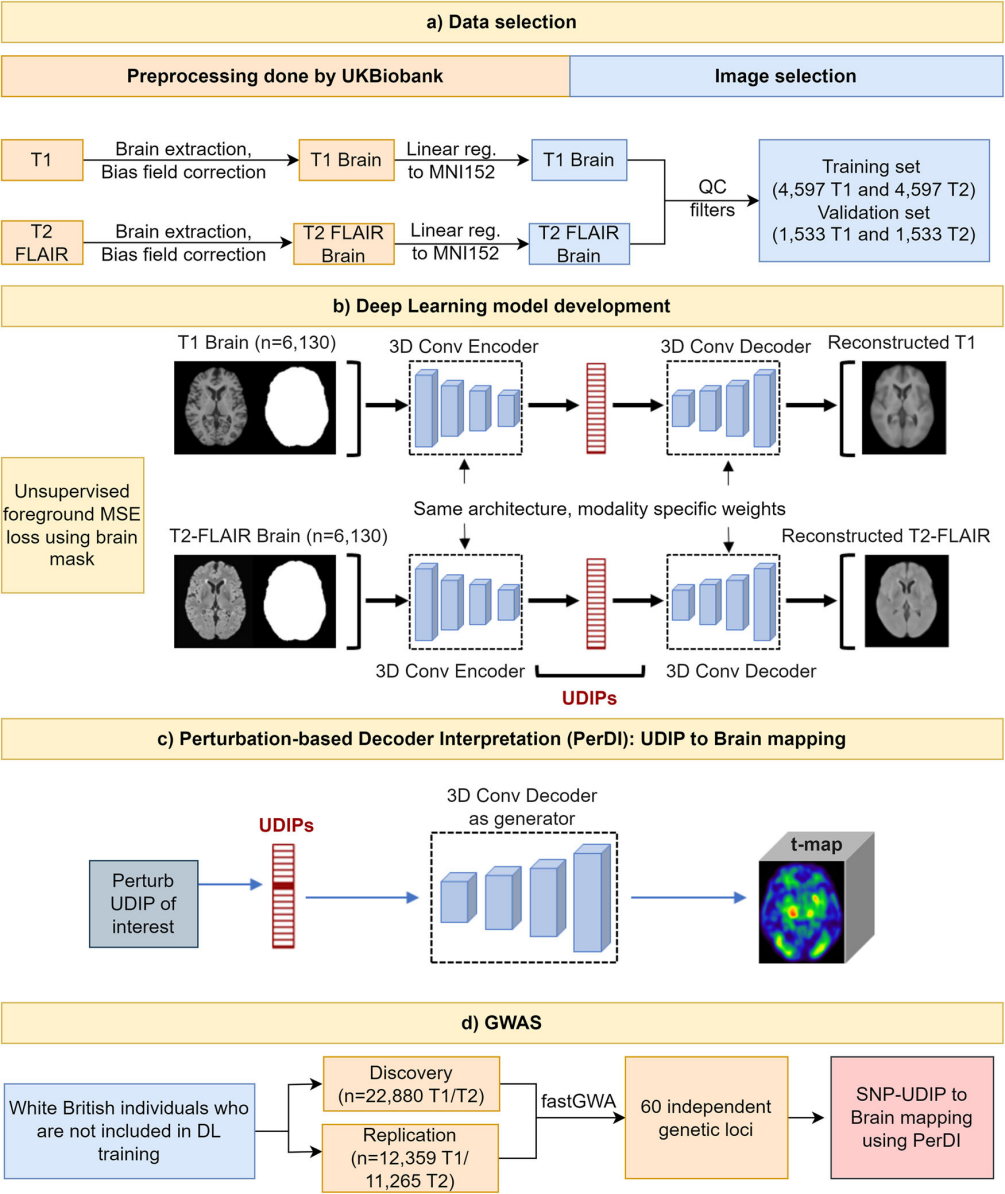
Overall pipeline of the study. **a** T1 and T2 FLAIR brain MRI preprocessed by UKBB were divided into separate datasets for deep learning model development and conducting GWAS. **b** The autoencoder architecture was trained by background masked mean square error (MSE) loss. **c** Perturbation-based Decoder Interpretation (PerDI) method was developed to map UDIPs to brain regions. **d** GWAS conducted using a discovery set identified 9,457 SNPs organized into 97 independent genetic loci out of which 60 loci were replicated in the replication set. PerDI was used to map SNP-UDIP pairs to brain regions.

The overall rationale of the study is to leverage the large sample size of UKBB brain imaging data to train an unsupervised deep learning model to generate brain imaging phenotypes and then conduct genetic association studies over UKBB data with both imaging and genetics data. Our overall analysis framework can be divided into four phases: data set selection, deep learning model development, GWAS, and interpretable deep learning model for mapping brain imaging phenotypes to brain regions (Fig. 1). Details of these phases are provided in the Methods section.

A total of 46,099 T1 (44,181 subjects) and 45,294 T2 (43,381 subjects) UKBB MRIs were downloaded. To avoid data leakage, we use disjoint data sets for model development and genetic association study. A dataset of 6130 images from subjects of mixed ethnicities was chosen as the model development set. This diverse dataset enables the model to learn a greater coverage of variabilities in brain morphology. GWAS was carried out on predictions generated by the model on a separate dataset of white British subjects consisting of 35,239 T1 weighted images and 34,145 T2 weighted images not included in the model development phase. GWAS was performed by dividing the subjects into discovery (22,880 T1 and 22,880 T2) and replication groups (12,359 T1 and 11,265 T2). A detailed data set selection process is shown in Supplementary Data 1.

To derive a compact representation of the input brain image, we use a 3D convolutional autoencoder. Autoencoders are a general architecture for deriving compact representations of any type of input object^26^. For 2D or 3D images, autoencoders with convolutional neural networks (CNN) architectures are a natural choice. While our architecture has semblance with the well-known U-net^27^, we do not introduce the skip connections between the encoder blocks and decoder blocks as we aim to retain maximal information through the bottleneck layer instead of generating sharp images at high resolution. Our architectural choices of the block with two 3 × 3 × 3 kernel for convolutional layers and one 2 × 2 × 2 kernel for max pooling are based on the fact that these choices are commonly used in many landmark convolutional neural network architectures inspired by the VGGNet and it has been shown to capture local spatial information effectively^27–31^.

We trained models with varying sizes and chose the one with 128 dimensions at the bottleneck layer, as it reaches a decent balance of model size and representation power. The training is effective as we see the difference between the original and the reconstructed images is much lower than that of random pairs (Supplementary Fig. 1c). Also, it is reassuring to see the reconstruction loss in the test set (GWAS set) is similar to that of the validation set even though they are from different ethnicities (Supplementary Fig. 1a, b). Although obtaining a high-quality reconstruction is not our primary goal, a visual inspection (see Supplementary Fig. 2 for examples of reconstructed images and Supplementary Fig. 3, 4 for lightbox views of original and reconstructed images) revealed that the reconstructed images share the general shape and anatomy of the original ones. However, many high-resolution features are not reconstructed due to the lack of skip connections to guarantee optimum data retention in UDIPs.

We performed single nucleotide polymorphism GWAS for each UDIP as a phenotype using linear mixed models. We identified 38,113 significant SNP-UDIP pairs with 9457 significant SNPs organized into 97 genetic loci with 126 lead SNPs in the discovery cohort (*p* < 5 × 10^−8^/256) (See Methods: GWAS-Loci clumping section). A total of 60 genetic loci were replicated in the independent replication set (*p* < 0.05/126). We identified 26 loci not found in traditional T1/T2 IDP GWAS of UKBiobank brain imaging phenotypes indicating the power of our approach for phenotype discovery^14,15^(Supplementary Data 2), although many loci were identified by later brain related GWAS studies. One of the loci on *PRDM1* gene was not previously indicated in any brain related GWAS.

### Characterization of the UDIPs

Both the 128-dimensional vectors for T1 and T2 are multivariate representations of the content of the input image. As we do not induce any structures among the 128 dimensions, we expect individual dimensions to be orderless and interchangeable. Indeed, we observed that all 256 dimensions are unimodal and normally distributed (Supplementary Fig. 5). Interestingly, there is a lack of general correlation (Supplementary Fig. 6) or subcluster structures (Supplementary Fig. 7) among them. This lack of correlation is not by design, but it indicates that from the totality of possible brain image information we have found 128 uncorrelated, independent dimensions. Also interesting is that the average absolute correlation within T1 UDIPs and T2 UDIPs (0.1031 and 0.1052, respectively) is smaller than that across T1 and T2 UDIPs (0.1128). This indicates that T1 and T2 embeddings each capture within-modality uncorrelated structural features while additionally capturing some overall features about the brain anatomy.

While it is challenging to interpret the 128-dimensional UDIPs directly, we verify that they capture relevant information. Using multiple linear regression analyses, UDIPs can accurately predict participants’ sex with the area under the ROC curve (AUROC) value for T1 of 0.9840 (0.0021) and T2 of 0.9781 (0.0001). This performance is comparable with existing literature for direct CNN-based sex prediction: 0.95 AUC using T1 and 0.92 AUC using T2^32^, 0.92 accuracy using T1 MRI^33^, 0.80 accuracy using T1^34^, and 0.99 accuracy using T1^35^. Also, age can be predicted from UDIPs with mean absolute error (MAE) of 3.3664 (0.0705) years for T1 and 3.1249 (0.0439) years for T2. Again, this performance is comparable to or better than existing CNN-based methods using T1 and T2 to predict age: MAE: 4.006 using T1 MRI^36^, MAE: 2.97 to 3.96 years using T2^37^, MAE: 2.14 years using T1 MRI^35^.

Compared with the traditional IDPs, the UDIPs are uniquely more informative: The UDIPs capture the overall shape and brain anatomy through reconstruction, which IDPs cannot. Still, we use multiple linear regressions to understand the linear correlations among the UDIPs and the FSL-derived volume IDPs. For predicting volumes of brain regions from UDIPs, the highest values of coefficient of determination (*R*^2^: T1/T2) were seen with volume of the following regions: ventricular cerebrospinal fluid (CSF) (0.9849/0.9625). This is due to the fact that in a typical T1/T2-FLAIR MRI, the ventricular CSF is the largest dark region inside the brain mask with a clear boundary, which is best captured by the MSE loss we used. Other regions of high prediction *R*^2^ are peripheral cortical gray matter (0.6993/0.6925), gray + white matter (0.7384/0.7242), gray matter (0.6928/0.6805), and thalamus (0.6664/0.6093) for both T1 and T2. On the other hand, for predicting the UDIPs from the volume IDPs, the highest R^2^ are about 0.568 for both T1 and T2 and the overall *R*^2^ values are not high. Complete list of *R*^2^ values can be found in Supplementary Data 3.

**Fig. 2:**
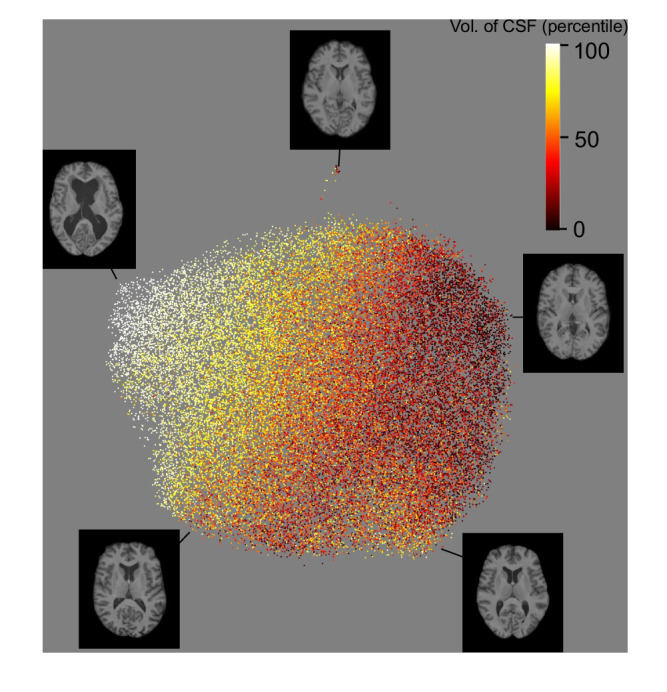
UMAP visualization of T1 UDIPs demonstrated their correlation with brain volume measures. UDIPs of 37,376 T1 images are reduced into two components using UMAP and colored with volume of ventricular CSF ranked as percentile. Axial slices of the T1 brain show variation in CSF as colored by UMAP and demonstrate the patterns captured by the UDIPs. x- and y-axes are arbitrary up to translations and rotations.

To visualize the population distribution of multivariate UDIPs, we used Uniform Manifold Approximation for unsupervised dimension reduction (UMAP)^38^ to reduce the 128-dimensional UDIPs into 2D (Fig. 2 and Supplementary Figs. 8–10). For UMAP, most participants’ UDIPs are distributed within a large continuous region, except some small groups of participants (24 T1 UDIPs, 18 T2 UDIPs) have UDIPs in isolated islands, probably due to having high volume of subcortical structures (Fig. 2, Supplementary Data 4). Also, unlike many embedding works in the literature, the goal of our representation learning is not to form clusters for downstream classification purposes but to uniformly encode the information about the imaging data^39^. Under this setting, clusters of representations are not desired and may even harm the GWAS performance due to reduced capacity for other characteristics. Therefore, the lack of clusters is a feature rather than a limitation of the approach. The UDIPs are clearly correlated with volume of ventricular CSF, consistent with the multiple regression analyses (Supplementary Data 3) and pairwise Pearson correlation (Supplementary Data 5). The correlations of UDIPs with age and sex are also visible (Supplementary Fig. 8). The components derived from UMAP for T1 and T2 UDIPs are correlated (Component 1 T1/T2: *r* = 0.38, Component 2 T1/T2: *r* = 0.77) (Supplementary Data 6). In addition, principal component analysis (PCA) (Supplementary Fig. 11) and t-distributed stochastic neighbor embedding (tSNE) (Supplementary Fig. 12) analyses of UDIPs show a similar separation of groups as UMAP. Of note, t-SNE seems to be capturing more local patterns. The reconstruction shows the shape, volume, texture, and anatomic relationship captured by the UDIPs.

Of note, the ideal dimension size for our UDIPs is not obvious. Rather than conducting a theoretical analysis, we took a data-driven approach. We trained four versions of UDIPs, of 32, 64, 128, and 256 dimensions, with otherwise identical encoder and decoder architectures, of T1 images and compared their representation powers through their reconstruction loss (Supplementary Data 7. a.). While increasing UDIP dimensions from 32 to 128 reduces the reconstruction loss, there is no obvious benefit in increasing the dimensions from 128 to 256. Also, we conducted a canonical-correlation analysis (CCA) among these versions of UDIPs and compared the variance of one version of UDIP that can be explained by another version of UDIP. Our findings indicate that the majority (>97%) of the variance in 32-dim and 64-dim UDIPs is explained by the 128-dim UDIPs, yet these lower dimensions only partially (<94%) elucidate the variance in 128-dim UDIPs (Supplementary Data 7a). Additionally, not much further increase in explained variance was achieved with 256-dim UDIPs compared to 128-dim UDIPs. As such, our selected dimension size of 128 ensures substantial representation while keeping reconstruction loss minimal, as validated by both the CCA analysis and the reconstruction MSE metrics.

### Interpretable model for mapping UDIPs to brain regions

To identify relevant brain regions for our UDIPs, we design and adopt a perturbation-based Decoder Interpretation (PerDI). This is because we are mainly concerned about how the changes to our UDIPs translate to the variability of brain MRI images. Briefly, for a UDIP dimension of interest, we generate perturbed reconstructions by adding one standard deviation to the dimension of interest of the UDIP vector of an input image. We use voxel-wise paired t-tests to compare the original and the perturbed and original reconstructions for 500 randomly selected brain images. For each UDIP, we generate a smoothed t-map to highlight its most relevant brain regions. See Methods: Decoder interpretation for a detailed description of our methods.

**Fig. 3:**
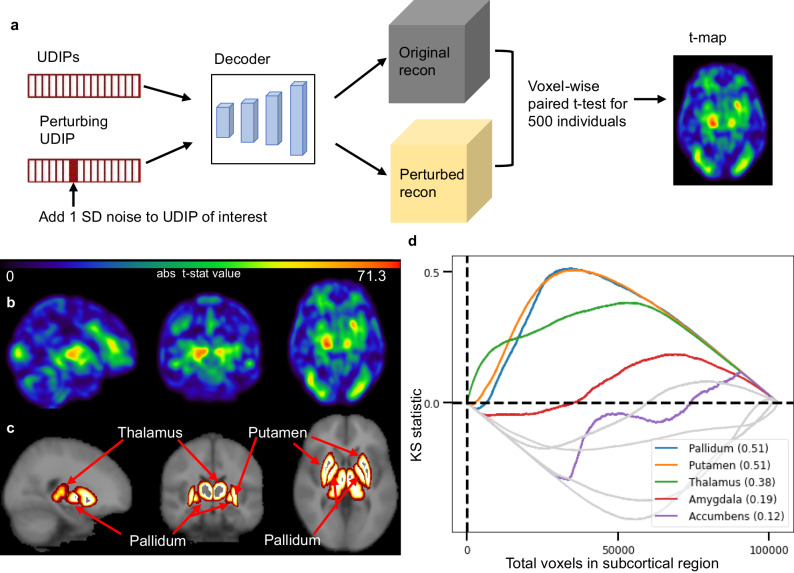
Perturbation-based decoder interpretation (PerDI). **a** One standard deviation is added to the dimension of interest in the UDIPs of one input image to generate a perturbed reconstruction image. The differentials of the perturbed and original reconstructions highlight the voxels relevant to the dimension of interest. We repeat this for 500 randomly selected images and use paired *t* tests to generate a voxel-wise t-map. **b** Absolute value of t-map generated for T2:67 by PerDI. **c** The Harvard-Oxford atlas labels for relevant subcortical structures. **d** Kolmogorov–Smirnov statistic is computed to identify subcortical regions of importance in the t-map generated by PerDI. K-S enrichment can highlight small regions that might not be prominently visualized in the t-map. Plot shows putamen (K-S: 0.51), pallidum (K-S: 0.51), and thalamus (K-S: 0.38) as the most prominent regions.

Although no brain segmentation and region annotation were used during training, some UDIPs hit on punctuated subcortical structures as quantified by the enrichment of high-ranking voxels in regions of interest (Fig. 3). We use brain structures in the Harvard-Oxford cortical and subcortical structural atlas to annotate prominent regions in t-map and use Kolmogorov-Smirnov (K-S) statistics to quantify the match of the brain structures (see Methods: t-map annotation). In general, the t-map of UDIPs are often not coincident to ROIs, as they are derived in an unsupervised fashion. We typically see single UDIP can span multiple atlas-defined brain structures with different weights. For example, UDIP 64 of T2 (T2:64) is found to represent multiple regions in the frontal lobe and lateral ventricles. T1 and T2 t-maps for individual UDIPs are made available on figshare repository^40,41^. K-S statistic values for all dimensions can be found in Supplementary Data 8.

Comparing the regional enrichment between all UDIPs and all brain regions (Supplementary Data 8), the UDIPs have decent coverage of most regions: For T1 and T2, 43 out of 47 cortical regions have some UDIPs with K-S statistic > 0.33 and 7 out of 11 subcortical regions have some UDIPs with K-S statistics > 0.40 respectively. See Supplementary Figs. 13–16 for maximum K-S statistics.

### Genetic association study of UDIPs

We conducted SNP variant GWASs for each UDIP as phenotype using linear mixed models over the array-genotyped markers. In the discovery phase, to adjust for multiple testing of a total of 256 UDIPs from both T1 and T2, the *p* value threshold is set to (5 × 10^−8^)/256. We identified 38,113 significant SNP-UDIP pairs with 9457 significant SNPs, from which 126 lead SNPs were identified, forming 97 genomic loci (Supplementary Data 2). Out of 126 lead SNPs, 71 passed the *p* value threshold of 0.05/126 in the replication cohort, replicating 60 independent loci (Supplementary Fig. 17, Supplementary Data 2). See Methods: GWAS for a detailed description of methods. We share individual Manhattan plots and QQ plots for each dimension at figshare repository^42^.

Overall, the genome inflation factor is well-controlled (Supplementary Fig. 18a). Mean LDSC intercepts for T1 and T2 are close to 1 (Supplementary Fig. 18b). All but one loci are reported with brain-related phenotypes (*p* < 5 × 10^−8^) according to the GWAS catalog (Supplementary Data 2, 9) (see Methods: Querying GWAS catalog). UDIP T2:121 also identified a unique locus on the *PRDM1* gene which was previously not indicated in any brain related GWAS. Notably, our method identifies 26 additional loci (Supplementary Data 2) that were not previously reported by the BIG40 study, which utilized all available conventional image-derived phenotypes (1,437 phenotypes) from T1 and T2-FLAIR brain MRI modalities^14,15^. We anticipate identifying loci that have not been identified by single IDP-based approaches due to the fact that UDIPs are not restricted to single, specific anatomical regions and can extend across multiple regions while encoding image reconstruction information.

Interestingly, most UDIPs have higher heritability with 0.253 ± 0.039 (T1:0.259 ± 0.032, T2: 0.247 ± 0.044) according to LD score regression (LDSC)^43^ (Supplementary Fig. 19), than that of the UKBB T1/T2-FLAIR based IDPs^15^ 0.176 ± 0.069 (See Methods: Genome-wide association study (GWAS)). Our estimate is based on the same set of markers and reference LD population and roughly the same sample sizes and thus should be a fair comparison, even though LDSC is known to underestimate the SNP-based heritability, compared to other population methods, like GCTA^44^.

We also utilized FUMA pipelines for gene annotation and functional enrichment. For T1 and T2 UDIPs, 196 and 362 genes were identified in the discovery cohort, using p-value threshold of 5e-8/128, respectively for each modality. Gene set enrichment tests revealed autism spectrum disorder, schizophrenia and other brain related GWAS catalog gene sets predefined at FUMA were significantly enriched. See Supplementary Note 1 and Supplementary Data 10–13 for detailed gene-based GWAS catalog analysis using FUMA.

While 128-dim UDIP has better representation power and captures more variances of other dimension UDIPs than lower-dimension UDIPs, it is not obvious that the extra brain structure information captured in 128-dim UDIP is heritable. To investigate this question, we conducted multiple GWAS using the T1 discovery cohort across three latent space dimensions: 32, 64, and 128. We found that 128-dim UDIPs identified more loci and more unique loci than lower-dimensional ones (Supplementary Data 7b).

### Meta-analysis

We conducted a sample size weighted fixed-effect meta-analysis of the discovery and the replication GWAS summary statistics using METAL^45^. With the enhanced sample size, a total of 95,061 significant (*P* < 5 × 10^−8^/256) SNP-UDIP pairs involving 19,617 SNPs clustered into 199 loci are identified (Supplementary Fig. 20). Using the criteria in Methods: Querying Big40 results, we found 145 loci not close to any loci of previous traditional T1 and T2 IDP GWAS^14,15^. Using the criteria in Methods: Querying GWAS catalog, we identified 29 new unique loci previously unrelated to any brain-related traits (see Supplementary Data 14). Deep learning derived UDIPs capture novel patterns as phenotypes combining multiple regions unlike traditional phenotypes allowing to identify more unique loci. For example, a unique locus on chromosome 5 with lead SNP rs6868292 (*p* = 9.02 × 10^−11^) in the intron of the *PLPP1* gene was found associated with UDIP, T2:93, which was previously not associated with any brain-related trait by GWAS. PerDI revealed that T2:93 captures information from combination of regions comprising of planum polare and supramarginal gyrus (ant and post division) as prominent cortical regions and pallidum, putamen, and thalamus as the prominent subcortical region (Supplementary Fig. 21).

### Genetic correlation

We conducted a genetic correlation analysis between the results obtained from the meta-analysis (discovery and replication cohort) and summary statistics for ten brain-related conditions (see Supplementary Figs. 22, 23) chosen by Elliott et al’s UKBB study^14^.

There are no significantly correlated UDIPs after Bonferroni correction (0.05/2560). However, a few nominally significant associations are noticeable: Attention deficit hyperactivity disorder (ADHD) showed the most correlated UDIPs (44 UDIPs) (*p* < 0.05) with absolute maximum genetic correlation value of 0.1811 (*p* = 0.0003). Other conditions with high absolute maximum genetic correlations are amyotrophic lateral sclerosis (0.3284, *p* = 0.0052), ischemic stroke (0.2967, *p* = 0.0014), major depressive disorder (0.2681, *p* = 0.0029), autism spectrum disorder (0.2425, *p* = 0.0003), bipolar disorder (0.2229, *p* = 0.0002) and Alzheimer’s disease (0.2366, *p* = 0.0183). See Methods: Genetic correlation for more details.

### Use UDIPs for interpreting brain GWAS results

**Fig. 4:**
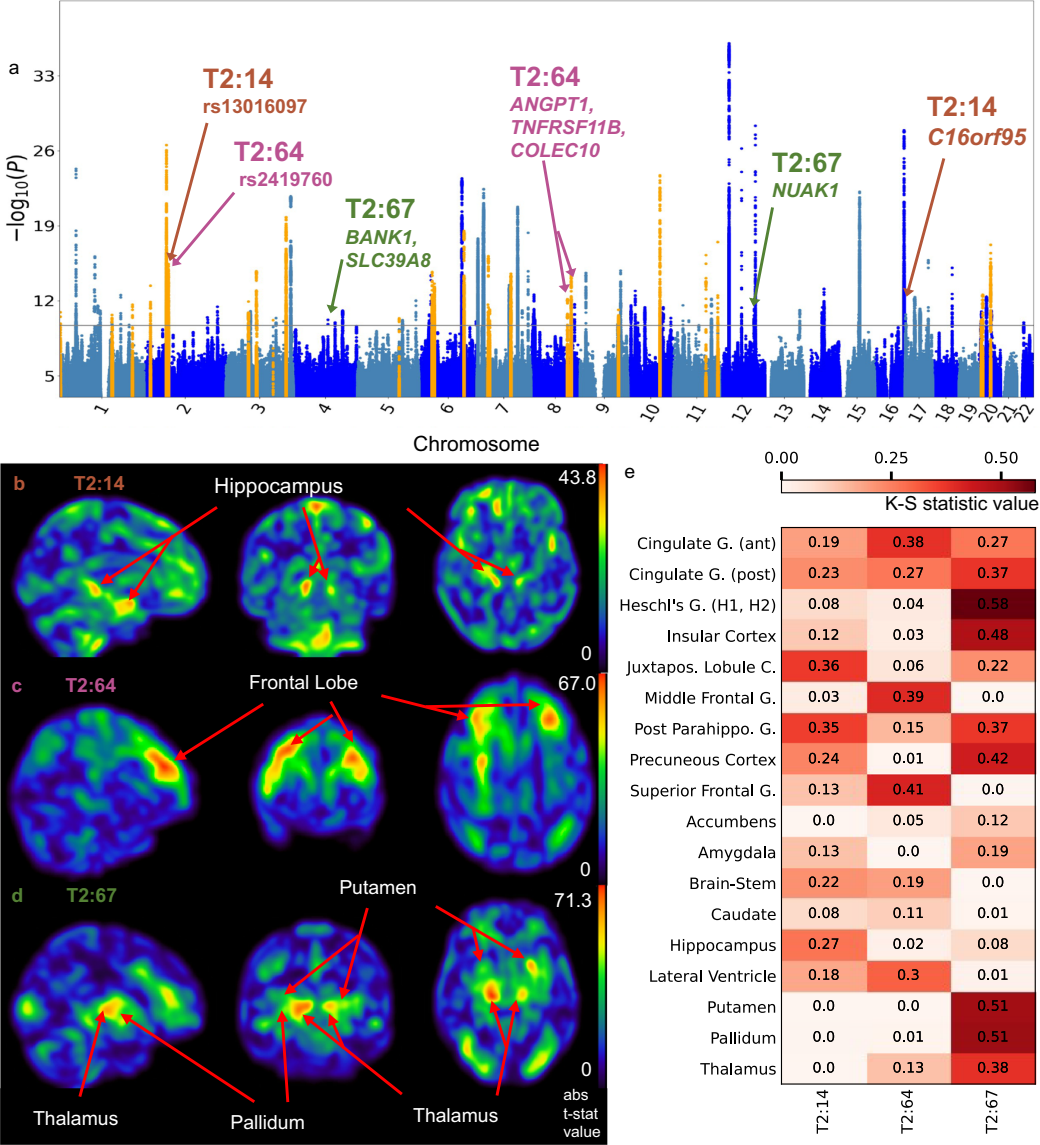
Perturbation-based decoder interpretation (PerDI) to interpret UDIPs associated with specific GWAS loci. **a** GWAS discovers and replicates loci identified by UDIPs T2:14, T2:64, T2:67. T2:14 identified loci on chr2 and 16, T2:64 identified loci on chr 2, 8, and 13, T2:67 identified locus on chr 4 and 7. **b** T2:14 t-map shows the hippocampus as the prominent region. **c** T2:64 t-map shows the frontal lobe as the prominent region. **d** T2:67 t-map shows the thalamus, putamen, and pallidum as prominent regions. **e** K-S statistic values for selected subcortical and cortical regions showing the regions represented by each UDIP. Acronyms: G Gyrus, C Cortex.

Effectively, our UDIPs can be a bridge between genetic variants and relevant brain regions, whereby providing anatomical details of a genetic association signal. For example, we found UDIP T2:67 is associated with rs13107325 (*SLC39A8*) (Fig. 4a). This SNP was previously found to be associated significantly (*p* < 5 × 10^−8^) with thalamic volume^46^, nucleus accumbens volume^8^, schizophrenia^47–50^, alcoholism^51–56^, intelligence^57,58^ and other brain morphology related traits (brain volume measurement, neuroimaging measurement, cortical thickness) in GWAS catalog. Association with Parkinson’s disease^59^ was reported with *p* = 7 × 10^−8^. We also found rs12146713 (mapped to *NAUK1* gene) associated with the same UDIP (Fig. 4a). rs12146713 was previously found to be associated with thalamus volume (medial thalamic nuclei volume)^46^, diffusion MRI derived white matter microstructure and integrity^15,60^, lateral ventricle volume^15^, cortical thickness and surface area, and subcortical structure volume^24^. These findings are consistent with our PerDI interpretation for this UDIP, which identifies putamen, pallidum, thalamus, amygdala, accumbens, and hippocampus as the main subcortical structures (Fig. 4d). Supplementary Figs. 24, 25 show K-S statistic plots for cortical and subcortical structures respectively for T2:67.

Of note, this locus was previously implicated in FSL and Freesurfer derived phenotypes (IDPs) to ventral caudate, putamen, ventral striatum, anterior cingulate cortex and cerebellar regions (Elliott et al.’s^14^ Extended Data Fig. 1). However, to visualize the MRI regions implicated by this SNP, the gray matter regions of MRI images of carriers and non-carriers were compared, which is not a cheap operation. Instead, the effect of this SNP to relevant brain regions can be efficiently visualized through the UDIP T2:67 (Fig. 4d).

One of the loci missed by previous UKBB IDP GWAS^14,15^ was identified by GWAS of UDIP T2:14 (Fig. 4a). The locus (chr 2) was previously found associated significantly (*p* < 5 × 10^−8^) with cortical thickness, cortical surface area, brain measurement, sulcal depth, and white matter microstructure in other brain imaging GWAS studies. We also identified another locus (chr 16) previously identified in UKBB IDP GWAS and associated with *C16orf95* gene. It is associated with brain age, periventricular white matter hyperintensities, CSF p-tau levels, lateral ventricular volume in normal aging along with similar traits as on locus on chr2. We identified Juxtapositional lobule cortex and parahippocampal gyrus as main cortical regions and hippocampus and brain stem as the main subcortical structures relevant to T2:14 (Fig. 4b). Supplementary Figs. 26, 27 show K-S statistic plots for cortical and subcortical structures respectively for T2:14.

UDIP T2:64 identified three loci on chr 2 and 8 (Fig. 4a) which were missed by UKBB IDP GWAS^14,15^. Locus on chr 2 is also identified by UDIP T2:14 described above. Two loci on chr 8 are associated with cortical thickness, sulcal depth, cortical surface area, white matter microstructure and education attainment in other brain imaging GWAS studies. In our study, UDIP T2:64 corresponds to portions of the frontal lobe (superior frontal gyrus, middle frontal gyrus, cingulate gyrus, frontal pole, precentral gyrus) and lateral ventricle as revealed using PerDI (Figs. 4c, e). This locus was missed by IDP GWAS possibly because IDPs were only descriptors of individual single regions while this UDIP captures a combination of features from the frontal lobe and the lateral ventricle. Supplementary Figs. 28, 29 show K-S statistic plots for cortical and subcortical structures respectively for T2:64.

A locus on *PRDM1* gene previously not indicated in any brain-related GWAS was identified by UDIP T2:121. It is seen mainly associated with frontal medial cortex and frontal pole in the cortical regions and hippocampus and lateral ventricle in the subcortical regions.

### Comparison of brain volume IDPs and UDIPs

Compared to traditional IDPs, our unsupervised-deep learning derived UDIPs are distinct in many ways (Table 1). We compare our GWAS results with BIG40 database of GWAS conducted by UK Biobank as they conduct GWAS using 1,437 traditional T1 and T2 phenotypes which covers all traditional approaches of defining phenotypes (https://open.win.ox.ac.uk/ukbiobank/big40/). Primarily, UDIPs are more heritable than IDPs. Individual UDIPs are more heritable than individual IDPs on average. Compounded on the fact that UDIPs are less mutually correlated than IDPs, this indicates that in total UDIPs capture heritable information in the brain more efficiently than IDPs. We postulate the possible reasons for UDIPs’ higher heritability might come from their better minimizing measurement error and reporting error, and ability to capture population variation. UDIPs, unlike IDPs, need minimal image preprocessing and are derived from whole-brain high-quality images without human labels reducing the chance of measurement error and reporting error. Moreover, while IDPs are derived after optimizing loss for each individual separately, UDIPs are derived after training on the entire population using random mini-batches, better capturing necessary total variation in the population to reduce loss. As a result, UDIPs have more flexible definitions of regions, could be featured capturing concerted changes combining traits from multiple regions, or only capturing changes in part of a region that is not present in other parts of the same region unlike IDPs that are limited to atlas-defined areas, allowing them to capture unique traits missed by IDPs.

**Table 1:** Comparison of image-derived phenotypes vs our unsupervised DL-derived imaging phenotypes

	IDP (1,437 descriptors of T1/T2 brain regions)	UDIPs (128 T1/128 T2 variables)
Derived	Software derived	Learned
Preprocessing	Heavy	Light (brain extraction, linear registration, bias field correction)
Heritability computed using LDSC^b^	0.176 ± 0.069	0.253 ± 0.039 (T1:0.259 ± 0.032, T2: 0.247 ± 0.044)
Power to reconstruction	No	Yes
Redundancy (average genetic-correlation)	0.169 (0.159)	0.144 (0.107)
Time required to derive traits	Hours	0.093 (0.00136) s^a^
Interpretation	Simple to interpret	Relatively difficult to interpret

^a^Time performance estimated from 10 runs of the complete script on 1000 unique individuals; computation time for a single subject averaged to ~0.093 s (SD = 0.00136 s)

^b^Only array-genotyped SNPs in autosomes are counted

Moreover, what makes UDIPs distinctive from IDPs is that rather than being passive descriptors, our UDIPs are active predictive encoding of the input image. Via the decoder, UDIPs allow us to reconstruct, albeit imperfectly, the original input. Although PCA or NMF are also optimizing some sort of reconstruction loss, their quality of reconstruction is not on par with UDIPs.

For training, rather than being generated via a feature-engineering process, our UDIPs are derived from feature-learning. In terms of efficiency, while IDPs are derived with heavy processing that takes hours, UDIPs are derived with minimal preprocessing and take seconds (on GPU though). Since our model’s checkpoint with trained model weights are shared, our results should be relatively easy to replicate.

## Discussion

We presented an unsupervised deep learning-based approach to capture complex patterns of the brain from the MRI to define phenotypes (UDIPs) for genetic association studies. Our 3D convolutional autoencoder neural network model was trained by a reconstruction loss function on 6130 full-sized T1 and T2-FLAIR (T2) weighted brain MRIs from UKBB. Using white British individuals not included in the deep learning training as the test set, we show that the 128 neurons of the bottleneck layer of the autoencoder as phenotypes can capture the shape and structure of the brain: analysis of UDIP using encoder-based reconstruction, unsupervised dimension reduction techniques such as UMAP, PCA, tSNE, and statistical methods such as regression showed that a vast amount of information in the input MRI image is captured, including brain’s volume, shape, texture, anatomy, and pathology. More interestingly, these UDIPs are more heritable than most traditional IDPs. GWAS of UDIPs identified 60 replicated loci, 59 of which were previously associated with brain phenotypes, establishing the validity of our approach. In a broad sense our UDIPs are derived from brain MRIs and with decent heritability and thus can be considered as endophenotypes. To make them endophenotypes in a strict sense, future work is needed to establish the connection of our UDIPs to illnesses^61^.

The use of deep learning phenotyping in GWAS for brain imaging can potentially uncover subtle, complex genotype-phenotype relationships that are not readily apparent with traditional phenotyping methods. In addition to revealing the genetic basis for brain structures of normal individuals, our approach can also potentially lead to better understanding of various neurological and psychiatric disorders. Furthermore, one of the most promising applications of this work is in the field of neurodegenerative disorders like Alzheimer’s disease. Alzheimer’s disease is a complex disease whose genetic architecture is still not well-understood. Neuroimaging endophenotypes like our UDIPs are expected to have a simple genetic architecture being direct measures of brain structure, are often measured more reproducibly and precisely in vast samples such as UK Biobank and the Alzheimer’s disease sequencing project, have better statistical power compared to disease status in case-control studies as seen in the number of studies^7,62,63^. Genetic variants associated with neurodegenerative diseases often have subtle effects on brain structure and function that can be captured better through the comprehensive imaging phenotypes obtained through our approach.

As a phenotype discovery approach, our UDIPs are patterns derived from a data-driven approach and capture concerted changes across the entire brain that frequently occur in the population. There are no explicit constraints to ensure a strict perpendicular dimension variation for autoencoders. However, the architecture and the training of autoencoders do encourage a low redundancy of the representation. First, the convolutional layers serve as an important component to reduce voxel-level redundancies. Instead of capturing voxel-wise values which can be highly correlated, applying convolutional kernels allows the autoencoder to capture higher-level patterns. Second, the training of the neural network is performed by reconstructing the given input. The training process enforces the model to learn a compact and efficient representation of the data that retains the most important information. This also encourages a low representation redundancy.

While our CNN autoencoder architecture has some resemblance to linear dimension reduction methods such as PCA, there are important differences. While linear methods can be kernelized to add nonlinearity, they are not as expressive as CNNs in general^26,64^. In fact, kernel methods can be seen as a special case of two-layer feed-forward neural network, where the hidden size is equal to the number of training samples^65^. This results in limited expressive capabilities, increased computation and storage costs when compared to deeper neural network architectures. Moreover, not all kernelized linear methods can be easily represented as an explicit embedding function that fulfills the purpose of deriving a phenotype. Secondly, the convolutional layers in our CNN autoencoder take advantage of the spatial locality characteristics inherent in imaging data, namely, two voxels that are close to each other exhibit higher dependency than those farther apart. Instead of treating each voxel separately and independently, the CNN autoencoder can capture important image priors^66,67^, making it more suitable for analyzing imaging data.

We show some UDIPs have good correlation with some anatomical structures and thus are more easily interpretable. These patterns go beyond the predefined single region volumetric features such as IDPs and thus interpretability is challenging for our UDIPs. While we attempted to address the interpretability issue in our PerDI approach, there is room for improvement. For example, there might be approaches that can enhance the interpretability of the UDIPs by introducing loss functions that encourage localization or conformation to predefined compartments.

Of note, recognizing the limitations of traditional single IDP-based approaches, post-hoc statistical approaches have been developed to extend single IDP GWASs to multi-IDP GWAS. Specifically, the MOSTest^24^ explicitly modeled multivariate omnibus brain measures associated with single SNP via multivariate statistical tests, yielding enhanced loci discovery. However, this method still requires the pre-selection and pre-computation of input variables, necessitating decisions about what to include and what to leave out, and incurring algorithmic limitations, like segmentation, inherent in image preprocessing. For example, the recent application of MOSTest to a set of 1284 cortical surface vertices^16^, was limited to polygonal representations inherent in Freesurfer, with all the ambiguities of computing a surface mesh, while failing to account for subcortical brain-genome associations. In other words, multivariate association of IDPs may attain more sensitivity than univariate GWAS by modeling multivariate combinations of the measures provided, but the power of such methods still depends on the quality of the individual measures. In a sense, our UDIPs can be viewed as a dictionary of nonlinear brain patterns, automatically and implicitly acquired by deep learning. These could themselves serve as input to multivariate association methods, with the vector of UDIPs as analogs of the array of IDPs in MOSTest, although we have not pursued this approach here because our main focus was phenotype discovery. However, the CNN-learned encoding implicitly extracts related visible brain features, analogous to IDPs, into each UDIP, without the need for selection or the inherent uncertainty of traditional algorithmic processing. This suggests that univariate GWAS with UDIPs is already a step ahead of IDP-based univariate GWAS and may also offer some advantages over multivariate methods that input pre-selected arrays of IDPs.

There are some limitations to our approach. First, the loss metric used for training our model, mean square error (MSE), smoothes the reconstructed images and loses the high-frequency signal. Moreover, higher contrast regions like ventricles tend to be better captured than inner regions with low contrast, such as white matter. Second, MRIs are linearly registered to preserve the overall shape of the brain. However, this has the consequence that the voxels across images are not perfectly aligned, which will affect the correspondence of UDIPs across individuals. We could study the effect of registration in future studies. Third, as the majority of the UKBB population is white British, we only use white British individuals for the GWAS, and thus the generalizability of our GWAS findings to other populations are not tested. Future GWAS of UDIPs on other populations are warranted^68^. Nonetheless, we used the multi-ethnicity samples for model training, which might help alleviate the population biases in terms of modeling. Fourth, our analysis was focused on the standard analysis pipeline of autosomes. Future investigation of the genetic factors on the X chromosome will help elucidate additional genetic architecture of the brain structure^68^. Finally, only internal validation using UKBB data is conducted. Future external validation in a different data set is warranted.

Our methodology offers a versatile framework that can be readily extended to various imaging modalities. For example, applying our approach to FA images from diffusion MRI could illuminate novel phenotypes representing white matter integrity of connections between brain regions. Beyond its application to brain MRI, our method can be adapted to work with other imaging types such as retinal fundus and OCT images, DEXA scans, and more.

While our UDIPs have higher heritability compared to traditional IDPs, our results showed that the majority of the loci identified by UDIP GWAS are already identified by IDP GWAS, including both the original BIG40 GWAS and the following up of brain imaging GWAS of UK Biobank and other data sets. Like many phenotypes, the genetic discovery of brain structure phenotypes is likely getting exhausted. Therefore, the primary challenge of the field is likely to transition into better characterization of the polygenic signals identified. Our work represents an attempt into this direction and more future works are warranted.

In conclusion, this work represents proof-of-concept of the application of an unsupervised deep learning approach as an imaging phenotyper for GWAS. Thus, it presents an innovation in the field, with the ability to automatically extract and interpret phenotypes from a diverse range of imaging modalities with minimal manual intervention. The method sets the stage for future research to uncover complex genotype-phenotype relationships, particularly in brain imaging.

## Methods

### Detailed dataset descriptions

UKBB is chosen because it is the largest public brain imaging study, and its data were uniformly processed with a standard pipeline. UKBB MRIs were downloaded on October 15, 2021. UKBB participants had birth year between 1934 to 1971 with female to male ratio of 0.52. UKBB^1^ has provided a bias-field-corrected version of the brain-extracted T1-weighted (T1) and T2-weighted FLAIR (T2) images captured mainly using standard Siemens Skyra 3T running VD13A SP4 (as of October 2015), with a standard Siemens 32-channel RF receive head coil. Resolution of T1 is 1 × 1 × 1 mm, and resolution of T2 is 1.05 × 1 × 1 mm (https://biobank.ctsu.ox.ac.uk/crystal/crystal/docs/brain_mri.pdf). To maximize the generalizability and minimize feature engineering, we followed a simple processing pipeline developed by the UKBB MRI team. UKBB mainly uses FSL to process brain MRIs (https://www.fmrib.ox.ac.uk/ukbiobank/). The main preprocessing done by UKBB involves defacing MRI, brain extraction (using FSL’s BET, and linear and non-linear registration to standard space using FSL FLIRT and FNIRT), and bias field correction (FSL FAST). Bias field corrected brain-extracted T1 and T2 are selected. All MRIs were linearly registered (affine registration with 12 DOF) to standard MNI152 space using the UKBB-provided pre-computed transformation matrix with FSL FLIRT^69^. Linearly registered MRIs were used to ensure the normalization of head sizes and the overall alignment of MRIs between different subjects while also preserving the structural deformation information in the MRI, unlike non-linear registration. Linearly registered, defaced, bias field corrected brain MRIs were used for all our analysis. Further, each affine registered MRI’s intensity was normalized using Z-score normalization by subtracting mean intensity and dividing the result by the standard deviation of the intensity. Background intensity was excluded in computing the mean and standard deviation of the MRI to prevent skewing toward zero. Background comprises the majority of the voxels and is later excluded in the loss calculation.

UKBB has also provided precomputed quality metrics for MRI like UKBB Data field 25735 “inverted contrast-to-noise ratio” and UKBB Data field 25,736 “Discrepancy between T2 FLAIR brain image and T1 brain image,” which were used to ensure the quality of the deep learning training set. Lower values in both metrics indicate higher quality. Images with values below 95 percentile for both the quality metrics were selected for deep learning training and validation set. Only one visit was kept if multiple visits were found to ensure uniformity in the dataset. A dataset of 6130 images was selected consisting of subjects of mixed ethnicities. The dataset was randomly divided into a training set of size 4597 images (75%) and a validation set of size 1533 images (25%). See Fig. 1 for the overall pipeline of the study. The validation set was used for tuning hyperparameters for model training and saving checkpoints. GWAS was carried out on predictions generated by the model on a separate dataset of white British subjects consisting of 37,376 T1 images and 36,231 T2 images not included in deep learning training. Those were further divided into discovery (22,880 for both T1/T2) and replication group (12,359 T1 and 11,265 T2) for performing GWAS. See Supplementary Data 1 for sample size description.

### Deep learning architecture

Deep 3D convolutional autoencoder was used to obtain the 128-dimensional phenotype (See Supplementary Fig. 30). A separate model was trained for T1 and T2. The architecture was implemented using PyTorch and trained with the PyTorch Lightning framework. To obtain representation of the whole brain, we take the full-resolution brain T1 and T2 MRI as input. The model consisted of an initial convolutional block, four encoder blocks, a linear latent space of 128-dimension, four decoder blocks, and a final convolutional block and has 138.12 million parameters. The initial convolutional block consisted of two blocks of a 3D convolution layer (Conv3d) of kernel size 3 and stride 1 followed by a 3D batch normalization layer (BatchNorm3d) and Leaky rectified linear unit function (LeakyReLU). Each encoder block comprises a 3D max pooling layer with kernel size of 2 followed by two blocks of Conv3d of kernel size 3, BatchNorm3d, and LeakyReLU. The linear layer converts the last encoder’s output into 128-dimension latent space without any spatial resolution. This bottleneck vector is the representation that we are interested in as the UDIPs. Afterwards, we use the same number of decoder blocks with deconvolutional layers that gradually increase the image resolution while reducing the number of channels. Each decoder block comprises two blocks of Conv3d with kernel size 3, BatchNorm3d, and LeakyReLU, followed by 3D transposed convolution operator with stride of 2 and kernel size of 2. The final convolutional block comprises two blocks of Conv3d with kernel size 3 and stride 1, BatchNorm3d, and LeakyReLU, followed by a 3D convolutional layer with kernel size 1. The output image is of the same size as the input MRI (182 × 218 × 182).

We make the following remarks on our design choices. First, we chose a standard convolutional encoder and decoder architecture as such designs are known to deliver good performance. Second, while our architecture has semblance of the well-known U-net^27^, we do not introduce the skip connections between the encoder blocks and decoder blocks as we hope to retain maximal information through the bottleneck layer. Since we are not aiming to generate sharp images at high resolution, the skip connections are not necessary. Third, we use the full image as input and each of the bottleneck neurons has the receptive field of the entire image. This design will ensure each UDIP can be a descriptor of any feature across the entire brain. Fourth, we use 128 dimensional vectors as UDIPs as they are providing a comprehensive description of the entire brain morphology while making GWAS computationally feasible. But a larger number of dimensions are possible.

Compared to most previous deep learning-based brain MRI studies, the scale of our work is large both in terms of image size and sample size. Although no previous studies used an unsupervised approach for obtaining imaging phenotypes for GWAS for brain, some studies used autoencoder-like architecture to derive features for disease predictions such as Alzheimer’s disease^70^, schizophrenia^30^, suicidal ideation prediction^71^, and Autism spectrum disorder^72^. However, due to computational constraints, common approaches either downsize the image by reducing resolution, leading to loss of detail information, or feed the images in patches, losing the panoramic view of the complete MRI that better encodes anatomical relationship, or filter the image by extracting only gray matter and thus losing the rest of the brain information. We used a linearly registered whole-brain MRI without splitting the brain into patches to derive imaging phenotypes and thus offer more complete encoding of the input 3D image. Leveraging on the large sample size of UKBB brain imaging study, we have sufficient sample sizes for training a model, as well as have sufficient sample sizes for GWAS.

### Deep learning training

The dataset was randomly divided into a training set of size 4597 images (75%) and a validation set of size 1533 images (25%). The validation set was used for tuning hyperparameters for model training and saving checkpoints. The checkpoint with the lowest loss in the validation set was used to generate UDIPs.

No activation function is used at the output, making it a regression task per voxel. As in the standard autoencoder, we use a reconstruction loss that will minimize the difference between the input and output images. Mean square error was used as the loss function. A mask was derived based on the input image where the background was excluded. The mean square error calculation included only the voxels corresponding to the brain region. Specifically, the loss was defined as ∑_*ijk*_(*R*(*i,j,k*) − *O*(*i,j,k*))^2^ *f*(*O*(*i,j,k*)), where *R* is the reconstructed imaging data, $O$ is the original imaging data, $f$ is the step function outlining the brain mask and $i,j,k$ are the spatial indices. Adam optimizer was used with an effectively tuned learning rate (Supplementary Note 2). Checkpoints were saved at the top 5 lowest validation loss. Both the models for T1 and T2 were trained for 75 epochs. Seven NVIDIA A100-SXM-80GB GPU cards were used for training. Each training and validation epoch took around 6 min.

### Characterization of UDIPs

Our deep learning-derived UDIPs are independent and can represent multiple regions of the brain. To better understand UDIPs, we used UKBB computed volume-related IDPs using standard imaging software suites such as FreeSurfer and FSL. We use IDPs from the following T1 categories: Regional gray matter volumes (FAST), Subcortical volumes (FIRST), T1 structural brain. We also use T2 field ID 25781 Total volume of white matter hyperintensities (from T1 and T2_FLAIR images). To better understand T2 UDIPs, we used the volumetric estimates calculated by UKBB from T1 as both modalities from the same visit have been registered by UKBB. All IDPs are normalized by head size. The analysis performed using UKBB IDPs include UMAP (Uniform Manifold Approximation for dimension reduction)^38^, PCA, t-distributed stochastic neighbor embedding, linear regression and logistic regression. Unsupervised dimension reduction techniques such as UMAP, PCA, t-SNE were used with default parameters. We used scikit-learn (v 0.24.2) for PCA and t-SNE and the UMAP python package (v 0.5.2) for UMAP. UKBB IDPs were used to color the 2D scatter plot of two components of each of the above approaches. For a continuous precomputed feature, we used rank based on percentile to make visualization possible. Sex is the only categorical feature. To better understand the deep learning derived imaging phenotypes, we used linear regression to predict IDPs from UDIPs. We use tenfold cross-validation over the test set which was set aside from the model training and validation sets. We keep only one visit for each patient to avoid data leakage. We use the mean coefficient of determination R2 from the tenfold as the metric. We also use MAE for age prediction from UDIPs to make it comparable with existing literature. We used logistic regression to predict sex from UDIPs and use area under the curve as the metric to make it comparable with existing literature.

We used CCA^73^ to understand the relation between information encoded by 128 dimensional UDIPs (X) and other dimensional UDIPs (Y: 32, 64 and 256 dimensions) to determine the ideal size of the latent space. Specifically, our implementation of CCA is based on singular value decomposition. We demean $X$ and $Y$ and then doing SVD on the demeaned data: $X={U}_{1}{S}_{1}{V}_{1}^{T},{Y}={U}_{2}{S}_{2}{V}_{2}^{T}.$ The canonical correlation $S$ is calculated from the SVD of ${U}_{1}^{T}{U}_{2}$: ${U}_{1}^{T}{U}_{2}={US}{V}^{T}$, variance of $X$ explained by $Y$ is $\frac{{\left|{S}_{1}U\odot {S}\right|}_{F}^{2}}{{\left|{S}_{1}\right|}_{F}^{2}}$ and variance of $Y$ explained by $X$ is $\frac{{\left|{S}_{2}V\odot {S}\right|}_{F}^{2}}{{\left|{S}_{2}\right|}_{F}^{2}}$, where $\odot$ denotes elementwise multiplication and $F$ denotes the Frobenius norm.

### Decoder interpretation

256 UDIPs learn representation all over the brain. To identify regions of the brain represented by a specific UDIP, we develop Perturbation-based Decoder Interpretation (PerDI). The regions of the brain of that specific UDIP can then be associated with the SNPs identified by the same UDIP. We add one standard deviation (σ) as noise to the specific UDIP we are trying to interpret while keeping other UDIPs constant. The original decoder is used to reconstruct images from the perturbed UDIPs (perturbed reconstructed images). The process is repeated for 500 MRIs from 500 randomly selected individuals for improving the robustness of the result. Paired t-test is carried out between the 500 original reconstructed images and 500 perturbed reconstructed images. Absolute t-map is obtained. Gaussian filter (*σ* = 3) is used to smoothen the final t-map. Using Gaussian blur reduces the impact of not using non-linear registration.

### t-map annotation

Harvard-Oxford cortical and subcortical atlas^74^ are selected to annotate the t-map. For each specific atlas, each voxel in the t-map is ranked from highest to lowest rank. For each region, normalized Kolmogorov–Smirnov test (K-S test) statistic is obtained. Specifically, the curve in Fig. 3d is defined as $\frac{k}{V}-\frac{n}{N}$, where $k$ is the number of voxels belong to a specific region in the top $n$ ranked voxels, $r$ is the ratio between the volume of the region and the whole brain, $V$ is the number of voxels in that specific region and $N$ is the total number of voxels. The higher the value of the K-S statistic, the higher the representation of the region by the specific UDIP. The brain regions corresponding to the UDIP can then be associated with the SNPs identified by the same UDIP through GWAS.

### Statistics and reproducibility

#### Acquisition of genetic data

The genome-wide scans were performed over UK Biobank’s imputed SNPs (Version 3) (UKBB Category 100319)^75^. This genome-wide genotype data was collected using Applied Biosystems UK BiLEVE Axiom Array and imputed using reference panels combining the Haplotype Reference Consortium and UK10K haplotype resources. The UK Biobank team had conducted quality control (QC) procedures, phasing, and imputation^75^.

#### SNP QC

After downloading the genetic data, we conducted the following additional SNP QC processing: After filtering by MAF > 0.0001 and missing rate <0.05, 8,931,083 SNPs remain. When conducting GWAS, an additional MAF > 0.0001 and missing rate <0.1 filtering was performed on each cohort, resulting in 8,925,988 SNPs in the discovery cohort and 8,925,870/8,925,814 SNPs in the T1/T2 replication cohorts. Hardy-Weinberg equilibrium test was not conducted: despite it is a standard practice for QC genotype data from directly genotyped markers, it may not be statistically sound to convert the dosage back to the best guess imputed genotypes and do HWE again.

#### Sample genotype QC

To control for potential confounding due to ethnicity, we only include white British participants (UKBB field ID 21000: Ethnic background and field ID 22006 (Genetic ethnic grouping)) who were not included in deep learning training for GWAS. We filtered out subjects with mismatched sex (field ID 31) and genetic sex (field ID 22001) and also subjects listed in the withdrawal list (as of 08/21/2023). White British participants (35,239 T1/ 34,145 T2) were split into discovery (22,880 T1/T2) and rest for replication cohorts. Further, to ensure independence between the discovery and replication samples, 457 subjects in replication with close relatives in the discovery cohort, defined by kinship coefficients > 2^(−4.5) = 0.0442, were removed. The final replication sample sizes are: 12,359 for T1 and 11,265 for T2.

#### Sample phenotype and covariate QC

To remove potential confounding effects, we included age (field ID 21003) a, a^2, sex (field ID 31) s, s x a, s x a^2, 10 genetic PC (field ID 22009), head size (field ID 25000), head position in scanner (field ID 25756-25758), scanner table position (field ID 25759), location of the assessment center (field ID 54) and date of attending assessment center (field ID 53) as covariates. The outliers in head size, head position and scanner position defined by median ± 5 standard deviations were removed. Additionally, inverted contrast-to-noise ratio (field ID 25735) was included in T1 GWAS as a quality indicator and similarly for discrepancy between T2 FLAIR image and T1 image (field ID 25736) in T2 GWAS.

#### Running GWAS

We used fastGWA^76,77^ from GCTA (Genome-wide Complex Trait Analysis) (Version 1.94.1) package for running GWAS using 256 embedding obtained from T1(128 dimensions) and T2(128 dimensions) MRI linear mixed model association analysis with a sparse kinship matrix provided by the UK Biobank. GWAS was run for both the discovery and the replication cohorts. For calling any SNP-UDIP pair genome-wide significant, we use a Bonferroni corrected p-value threshold of 5e-8/256 for the discovery cohort.

#### Post-GWAS QC

No genetic information was used while training the deep learning models which resulted in well well-controlled genomic inflation factor (Supplementary Fig. 18a).

The genomic inflation factor was calculated by dividing the median chi-square statistics by the inverse cumulative distribution function of chi-square distribution of 1 degree of freedom at 0.5. We used the LDSC v 1.0.1(https://github.com/bulik/ldsc)^78^ to calculate the intercept value as additional QC.

#### Loci clumping

We follow FUMA’s SNP2GENE^79^ protocol for loci clumping. Specifically, in order to clump the significant SNPs (*P* < 5e-8/256) into genomic loci, we first aggregated the 256 single UDIP GWAS summary statistics into a single summary statistics file by only taking the most significant *p* value among all 256 UDIPs for each SNP, also known as the “minP” approach. Afterwards, a two-step pruning process was performed. Significant SNPs were first pruned at LD *r*^2 = 0.6 to obtain a list of independent significant SNPs. The independent significant SNPs were further pruned at LD *r*^2 = 0.1 to identify the independent lead SNPs. A genomic locus was defined as the smallest contiguous region that contains all SNPs (including both the GWAS markers and the markers in the 1000 Genomes reference panel passing MAF threshold) with an *r*^2 value greater than 0.1 with the lead SNPs. If the physical distance between adjacent loci was less than 250 kb, they were merged together. Therefore, it is possible to have more than one lead SNPs per locus. All loci clumping analyses were conducted by FUMA^79^.

#### Replication

For replication, we run tests on a per-lead SNP basis. We then claimed a locus as replicated if any of the peak phenotype/variant pairs had *P* < 0.05/(number of lead SNPs) in the replication cohort.

#### SNP heritability estimate

We used the LDSC v 1.0.1(https://github.com/bulik/ldsc)^78^ to calculate the SNP heritability of both UDIPs and UK Biobank’s traditional IDPs using the same settings. The LD scores were calculated from 1000 Genomes. All these values were computed from the discovery cohorts, both having sample sizes of about 22k. The summary statistics of IDPs were downloaded from UKBB BIG40 server (https://open.win.ox.ac.uk/ukbiobank/big40/release2/stats/*.txt.gz).

### Querying GWAS catalog

We used FUMA to query candidate SNPs in each locus in GWAS to identify previously reported associations. FUMA directly calls GWAS catalog API and thus our results reflect the GWAS catalog as of September 2023. Candidate SNPs are defined by having *r*^2 > = 0.6 with independent significant SNPs (See Methods: Genome-wide association study (GWAS)) not only in the GWAS variants but also in the 1000 Genomes reference panel. We filter GWAS catalog results to include those with *p* value < 5e-8. We identified brain related traits from the GWAS catalog results using careful manual inspection.

### Comparison of brain volume IDPs and UDIPs

For Table 1, we compare our GWAS results with BIG40 database of GWAS conducted by UKBB as they conduct GWAS using 1437 traditional T1 and T2 phenotypes which covers all traditional approaches of defining phenotypes (https://open.win.ox.ac.uk/ukbiobank/big40/). The 1437 traditional descriptors of T1 and T2-FLAIR obtained through FSL and FreeSurfer can be divided into categories such as ‘Regional gray matter volumes (FAST)’, “Subcortical volumes (FIRST)”, “Freesurfer ASEG”, “Freesurfer BA exvivo”, “Freesurfer a2009s”, “Freesurfer DKT”, “Freesurfer desikan gw”, “Freesurfer desikan pial”, “Freesurfer desikan white”, “Freesurfer subsegmentation” and “volume of white matter hyperintensities”. For identifying unique loci not discovered in the BIG40 study, we processed the discovery summary statistics using the *p* value threshold of 5e-8/1437 to get a list of genomic loci (see Methods: GWAS), added 125 kb to both side of each locus and built an interval tree for each chromosome. We then processed our summary statistic the same way and queried the interval trees for overlapping. If an interval does not overlapping with the loci from the BIG40 study, the locus represented by this interval is unique and is at least 250 kb from any locus discovered in the BIG40 study.

### Meta-analysis

We used METAL(generic-metal-2011-03-25)^45^ to perform meta-analysis of GWAS summary statistics from the discovery and replication cohorts. We used sample size as the weighting factor in METAL.

### Genetic correlation

We used LDSC v 1.0.1(https://github.com/bulik/ldsc)^78^ to calculate genetic correlation and LD score from the meta-analysis results. We computed genetic correlations for ADHD, Alzheimer’s disease (AD), amyotrophic lateral sclerosis (ALS), autism spectrum disorder (ASD), bipolar disorder (BIP), ischemic stroke (IS), major depressive disorder (MDD), neuroticism, schizophrenia (SCZ), and sleep disorder (SD).

### Ethics oversight

Our analysis was approved by UTHealth committee for the protection of human subjects under No. HSC-SBMI-20-1323. UKBB has secured informed consent from the participants in the use of their data for approved research projects. UKBB data was accessed via approved project 24247.

### Supplementary information

Supplementary Information File

Description of Supplementary Materials file

Supplementary Data 1

Supplementary Data 2

Supplementary Data 3

Supplementary Data 4

Supplementary Data 5

Supplementary Data 6

Supplementary Data 7

Supplementary Data 8

Supplementary Data 9

Supplementary Data 10

Supplementary Data 11

Supplementary Data 12

Supplementary Data 13

Supplementary Data 14

Supplementary Data 15

## Data Availability

All results are available at http://deependo.org, figshare repositories^40,42,80^ and supplementary data. The GWAS summary statistics generated in this study are available at GWAS Catalog https://www.ebi.ac.uk/gwas/ with study accession ID GCST90319854. Data and code to generate Fig. 3d is provided in Supplementary Data 15.

## Code availability

We share our code and model checkpoints at https://github.com/ZhiGroup/DeepENDO and figshare repository^80^.
