# Supplementary material for: Unsupervised deep representation learning enables phenotype discovery for genetic association studies of brain imaging
Source: Commun Biol. 2024 Apr 5;7:414. doi: 10.1038/s42003-024-06096-7 (PMC10997628; doi:10.1038/s42003-024-06096-7)
Supplement: Supplementary file 1 — Supplementary Information File [file 42003_2024_6096_MOESM1_ESM.pdf]

Supplementary Materials of Unsupervised Deep Representation Learning Enables Phenotype Discovery for Genetic Association Studies of Brain Imaging

1. **Supplementary Notes**
2. **Supplementary Figures**

Supplementary Notes

Supplementary Note 1: Gene-based GWAS catalog analysis using FUMA

Rather than focusing on individual GWAS loci, we used FUMA pipelines to annotate genes and conduct functional enrichment of genes for UDIPs' GWAS summary statistics. For T2 UDIP, first, by the positional gene prioritization pipeline (gene assignment pipeline) from FUMA, a total of 362 genes were found (Supplementary Data 10). Using the Gene2Func pipeline of FUMA which performed Gene set enrichment analysis (GSEA), we identified 261 gene sets (adjusted p-value <0.05) using 20260 background genes (protein coding genes) (Supplementary Data 11). To better understand which phenotypes are implicated by our UDIP, we further analyzed the gene set enriched with various phenotypes of the GWAS Catalog used in FUMA. Out of all the phenotypes, the Brain morphology (MOSTest) gene set was the most significantly enriched gene set with an adjusted p-value of $1.28X10\text{-}^{138}$. Additionally, gene sets from 32 other brain-related phenotypes from GWAS catalogs were also significantly enriched, including for example, Subcortical volume (MOSTest) (adjusted p-value =$1.31X10^{-64}$), Subcortical volume (min-P) (adjusted p-value = $1.21X10^{-56}$), General factor of neuroticism (adjusted p-value = $4.8X10^{-18}$), and Parkinson's disease (adjusted p-value = $1.51X10^{-5}$).

The same procedure was followed for T1 UDIPs, and 196 genes were found (Supplementary Data 12). Three hundred forty-four gene sets were enriched (Supplementary Data 13). Overall, more brain-related phenotypes were associated with T1 UDIPs than T2 UDIPs (25 vs. 20). Similar to T2 UDIPs, *Autism spectrum disorder or schizophrenia-related* gene sets were the most significant gene sets. But more brain structure-related phenotypes like Hippocampal subfield CA4 volume, Total hippocampal volume, Hippocampal subfield CA3 volume, and Hippocampal subfield CA1 volume are enriched for T1 UDIPs. See **Gene annotation** for more information.

Gene annotation

FUMA[1] was used to annotate our GWAS results functionally from February 26, 2022, till April 15, 2022. FUMA has integrated the functional annotation pipeline, including SNP annotation, gene mapping, and gene set enrichment analysis. We used the summary statistics of GWAS as input and identified the prioritized genes through functionally annotated variants. We used the default setting in the FUMA web interface to find positionally prioritized genes within a 10 kb window of functionally associated variants. To assess the functionally enriched phenotypes associated with our UDIPs, we performed gene set enrichment analysis in FUMA with all unique genes identified in our UDIPs.

Supplementary Note 2: Learning rate for deep learning training

We used Adam optimizer with an initial learning rate (lr) of 0.0005248074602497723 for T1 and 0.0003019951720402019 for T2 was used with a batch size of 62. Learning rate finder (LR finder) function of the Pytorch Lightning framework was used to obtain the initial optimal lr. LR finder increases lr after each mini-batch and plots a lr vs loss plot. The plot is then used to select optimum initial lr. Learning rate was reduced by half when validation set MSE loss plateaued for four epochs with a lower cap of lr/1000.

Supplementary Figures

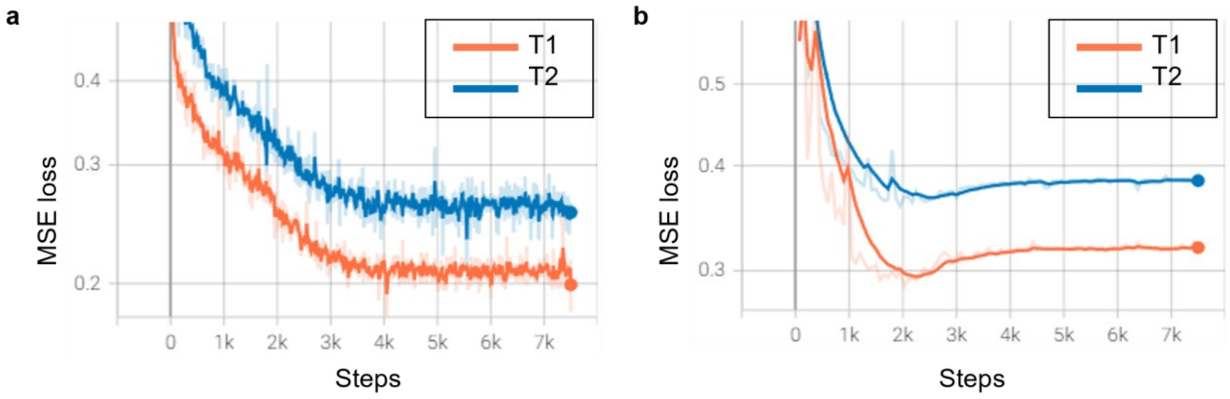

c

**Modality**	**Train** (T1:n = 4,597, T2:n = 4,597)	**Validation** (T1:n =1,533, T2:n =1,533)	**Test** (T1:n=37,376, T2:n=36,231)	**Random pairs of original and reconstructed images in test set**
**T1**	0.250 (0.022)	0.291 (0.033)	0.295 (0.032)	0.528 (0.106)
**T2 FLAIR**	0.322 (0.038)	0.360 (0.046)	0.362 (0.045)	0.669 (0.124)

Supplementary Figure 1. Voxelwise mean squared error (MSE) (squared L2 norm) loss focusing on the brain while masking out the background.
Test set is not included in the deep learning training and is used for defining discovery and replication cohorts for GWAS. a) Training loss curve b) Validation loss curve. Model weights corresponding to lowest validation loss were selected c) MSE loss for train, validation, and test set. MSE loss between random pairs of original and reconstructed images in the test set shows the model is learning brain morphology specific to individuals.

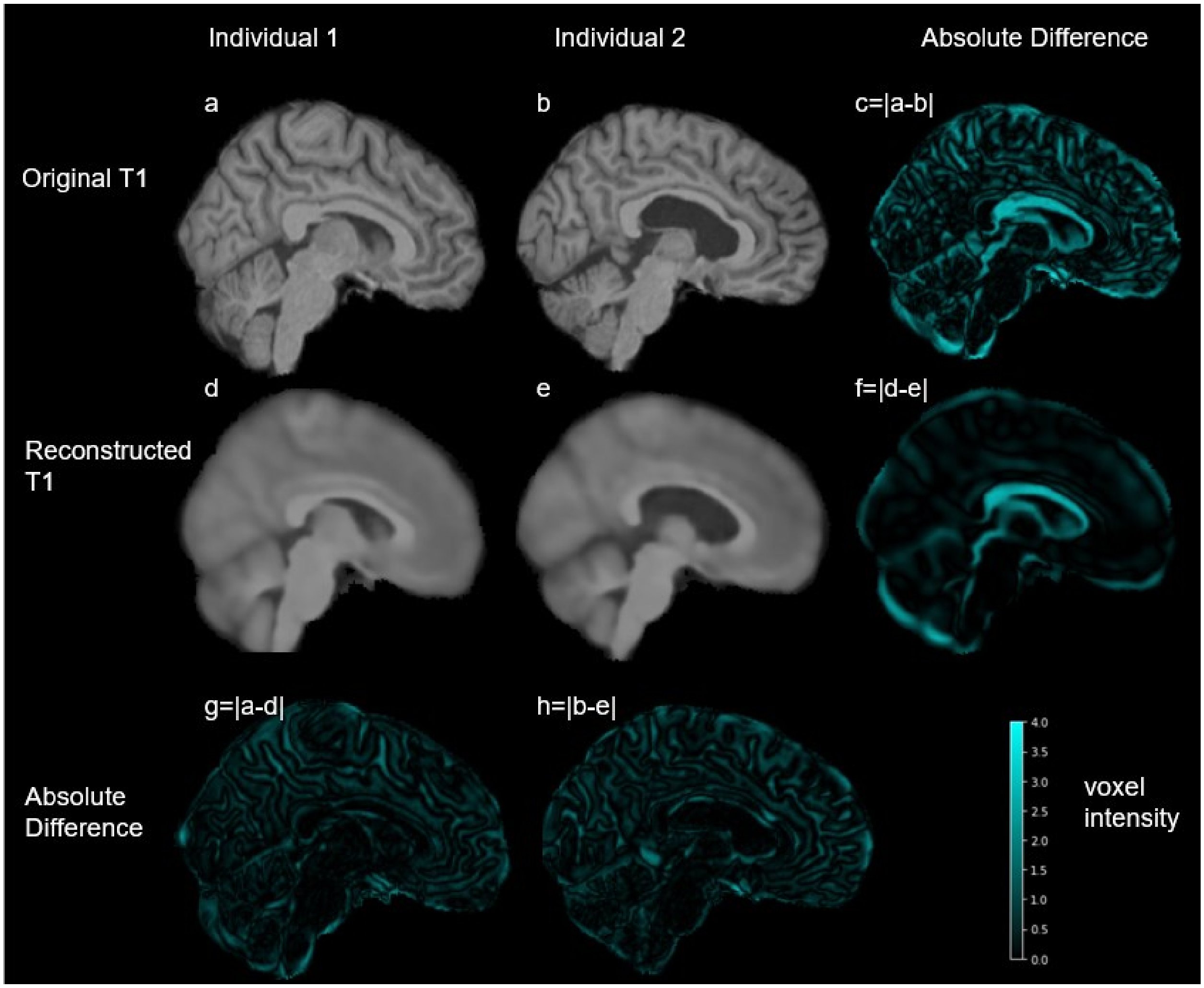

Supplementary Figure 2. UDIPs capture variations between individuals.
Original (a,b) and reconstructed (d,e) MRIs from two randomly selected individuals are shown. High frequency information may be lost in reconstruction due to absence of skip connections between encoder and decoder to maximize information retention in UDIPs. However, most of the overall structural details are retained. Absolute difference between two original T1 is shown (c=|a-b|) and absolute difference between two reconstructed T1 (f=|d-e|) are much greater between two individuals than between the original and the reconstructed MRIs for each individual (g=|a-d|,h=|b-e|). Model captured variance in morphology between individual subjects. Lightbox view representing multiple axial view slices in Supplementary Figure 3 (T1) and Supplementary Figure 4 (T2-FLAIR).

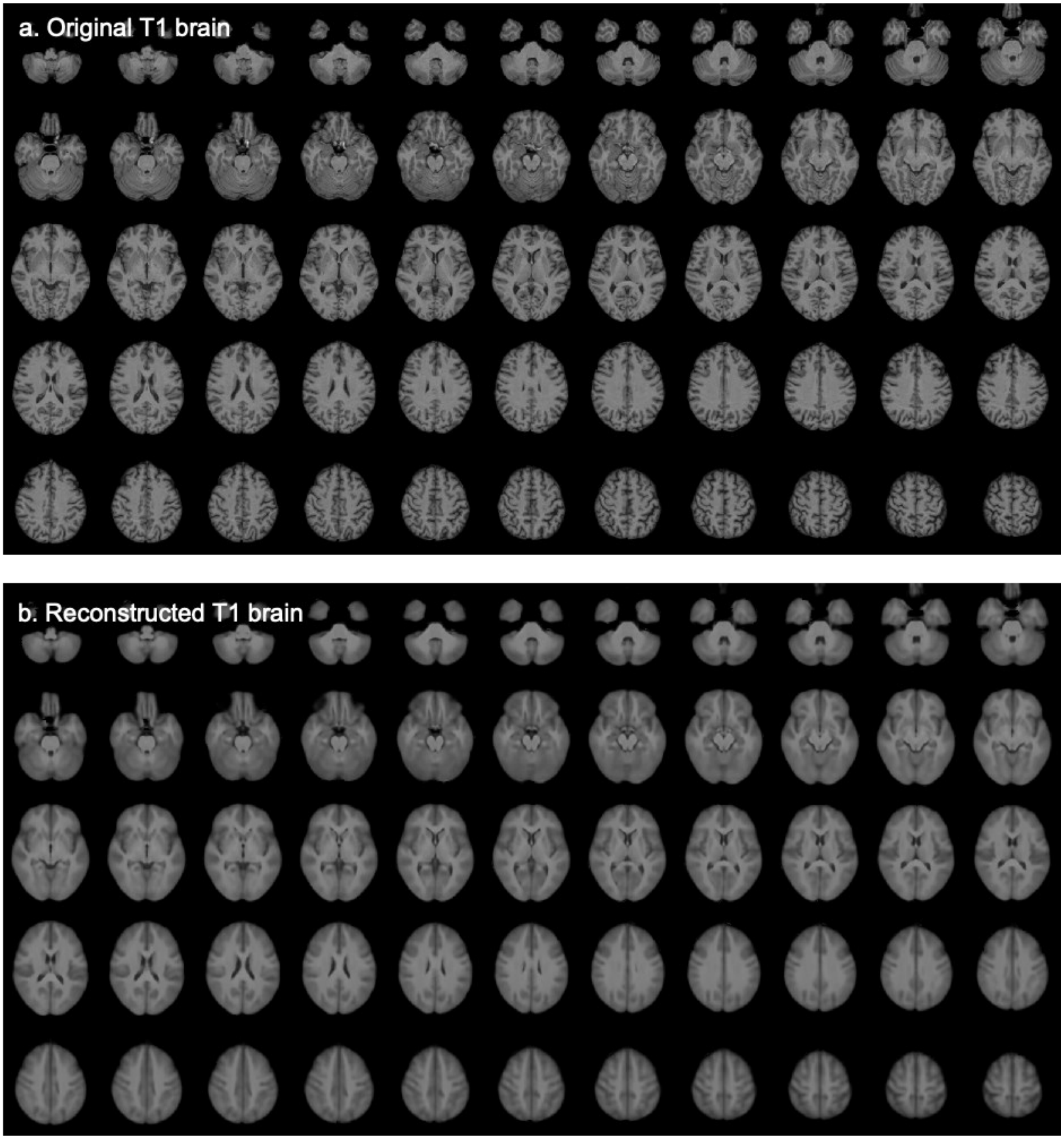

Supplementary Figure 3: Comparative axial views of original and convolutional 3D autoencoder reconstructed T1 MRIs demonstrating effective brain structure reconstruction from 128-dimensional UDIPs

Comparison between axial view of a) Original T1 MRI and axial view of b) Reconstructed T1 MRI shows that the convolutional 3D autoencoder was able to reconstruct the overall structural details of original T1 brain from the 128-dimensional UDIPs.

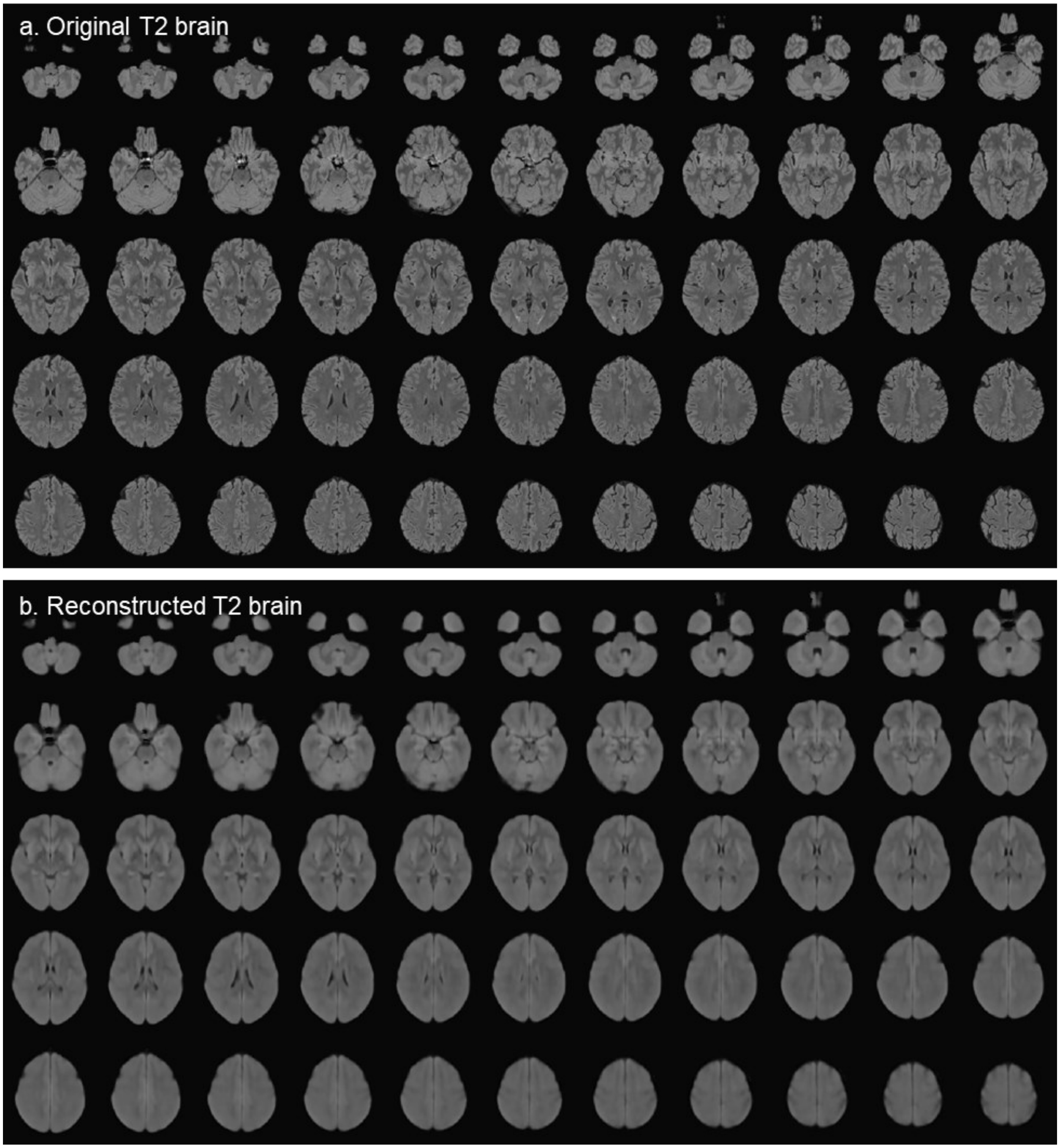

Supplementary Figure 4. Comparative axial views of original and convolutional 3D autoencoder reconstructed T2-FLAIR MRIs demonstrating effective brain structure reconstruction from 128-dimensional UDIPs

Comparison between axial view of a) Original T2-FLAIR MRI and axial view of b) Reconstructed T2-FLAIR MRI shows that the convolutional 3D autoencoder was able to reconstruct the overall structural details of original T2 brain from the 128-dimensional UDIPs.

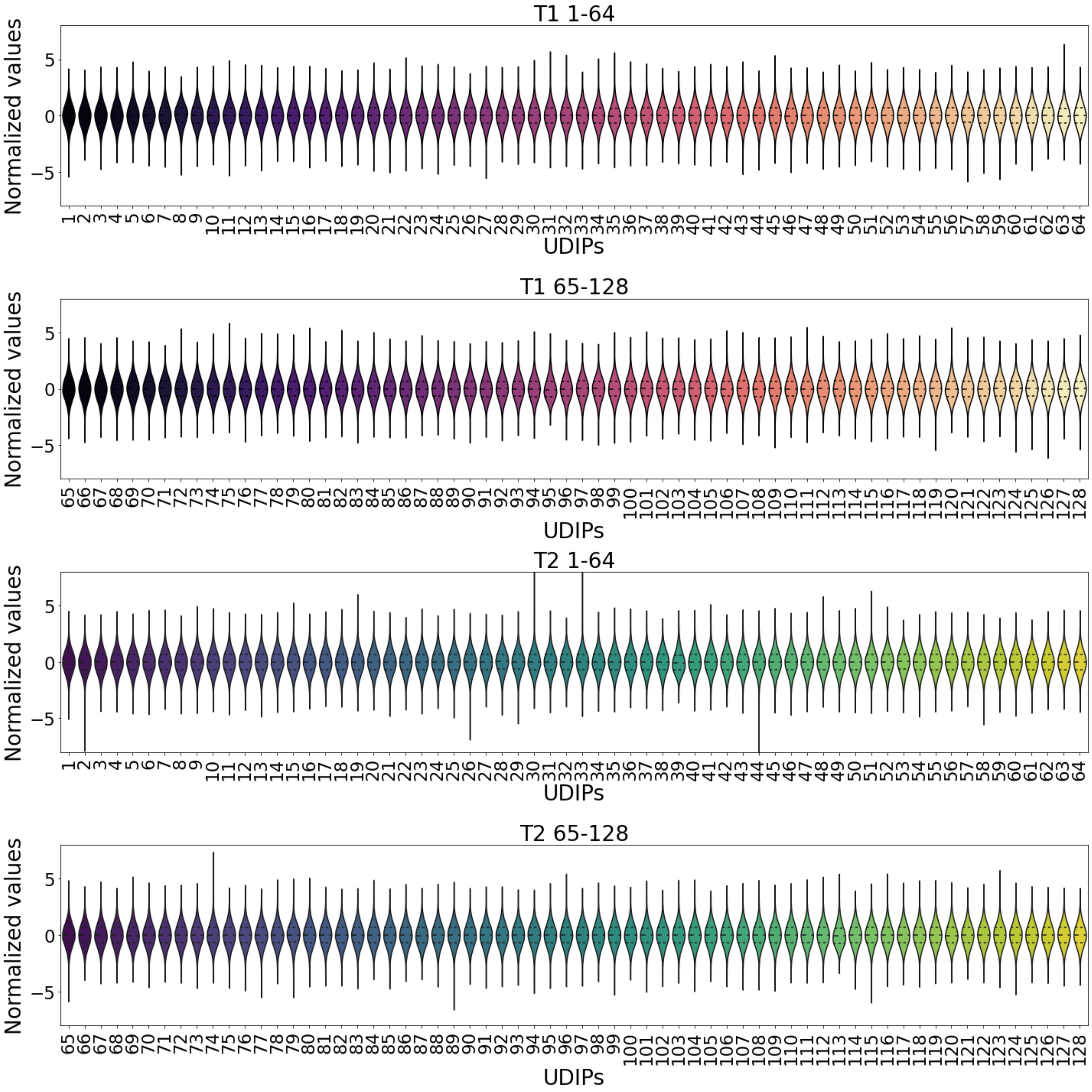

Supplementary Figure 5. Aggregate visualization of violin plot of 256-dimensional UDIPs showing normal distribution of each dimension.

It visually represents the distribution of normalized values for each UDIP. Within each plot, three horizontal lines indicate: bottom line as first quartile (Q1), middle line as median, and top line as third quartile (Q3). Width indicates the density of data points. These distributions collectively highlight the underlying normality of the UDIPs.

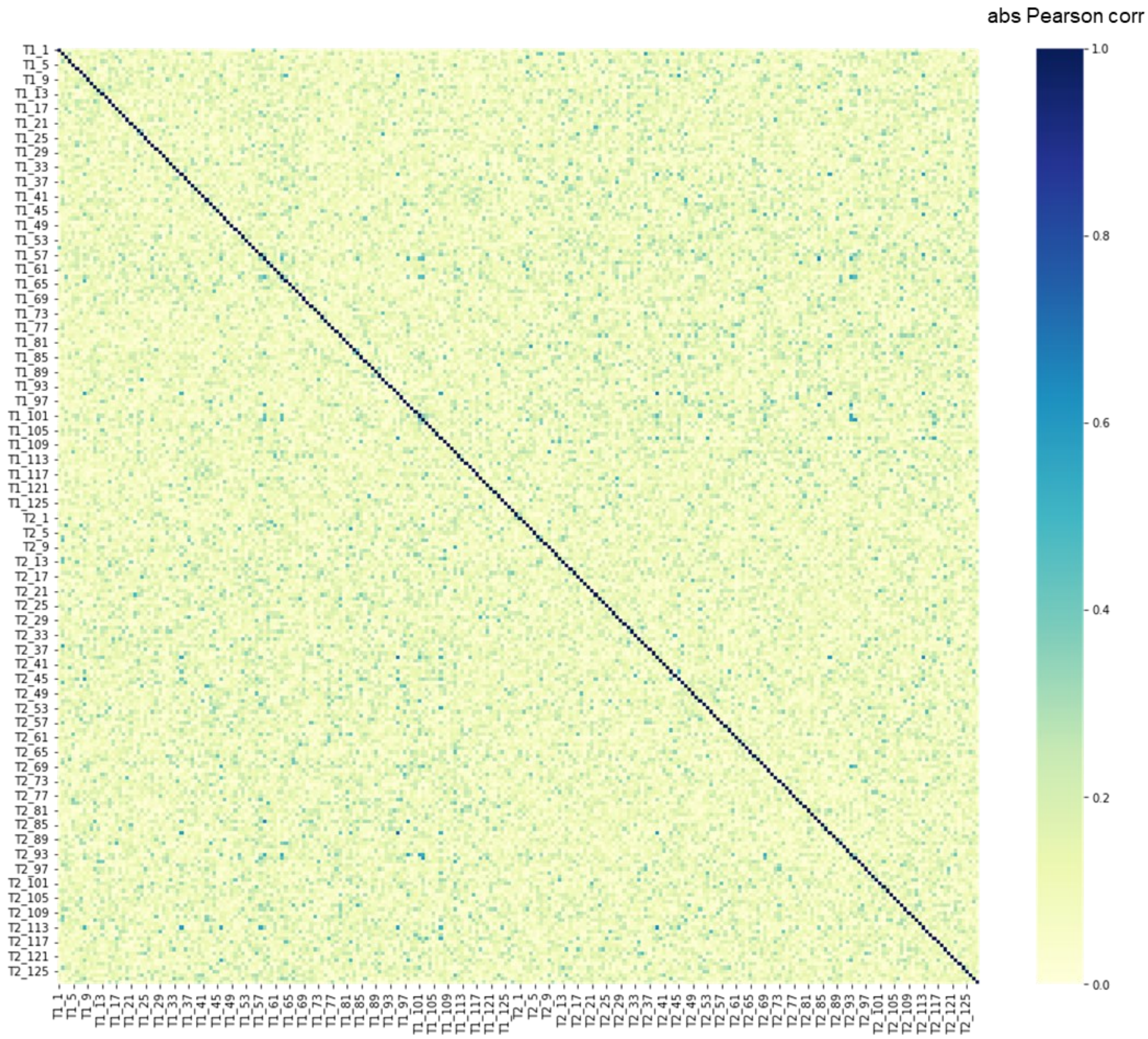

Supplementary Figure 6. Correlation heatmap using absolute values of Pearson correlation showing independence of the UDIPs.

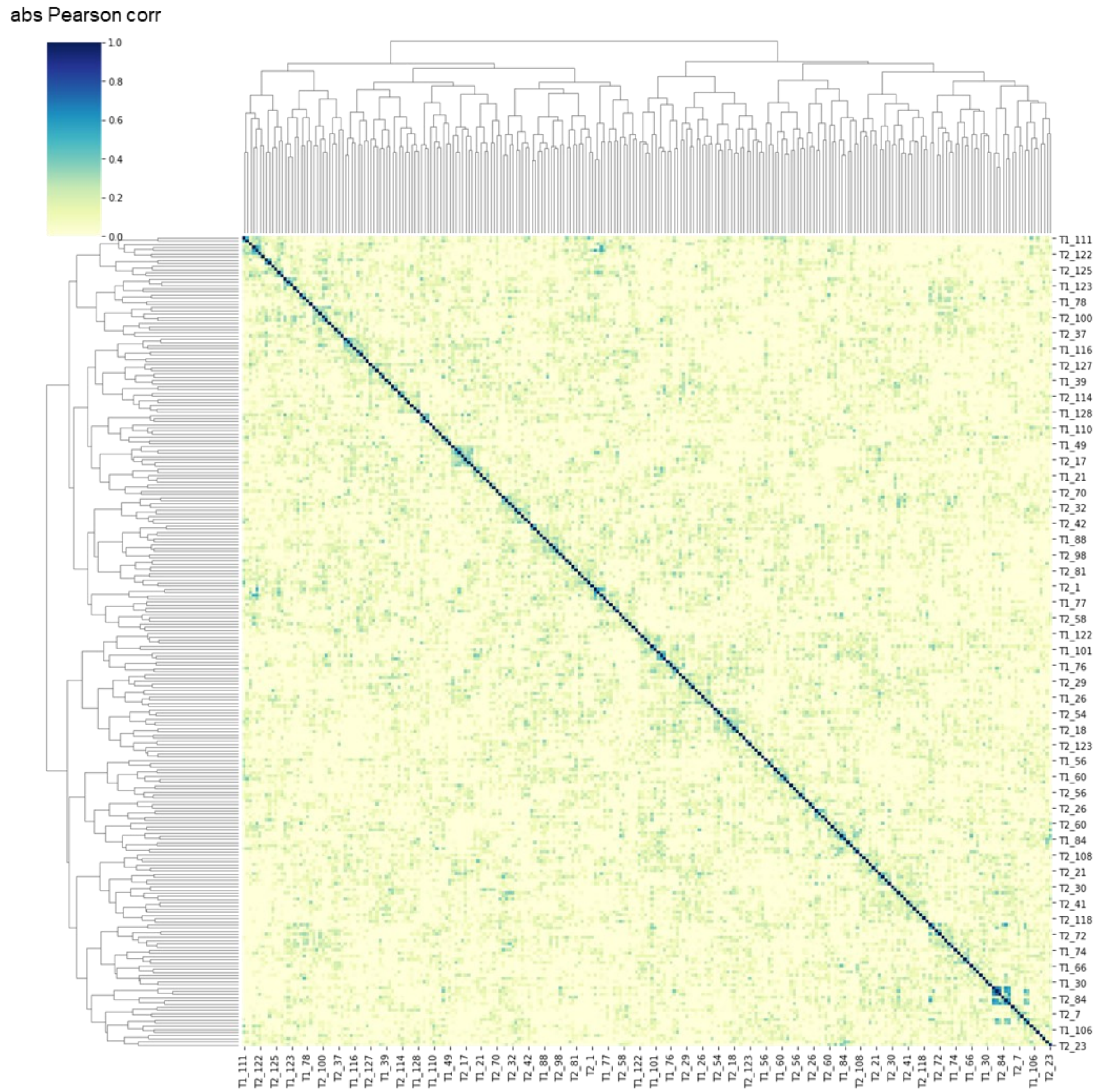

Supplementary Figure 7. Correlation heatmap with hierarchical clustering using absolute values of Pearson correlation showing lack of clustering of the UDIPs.

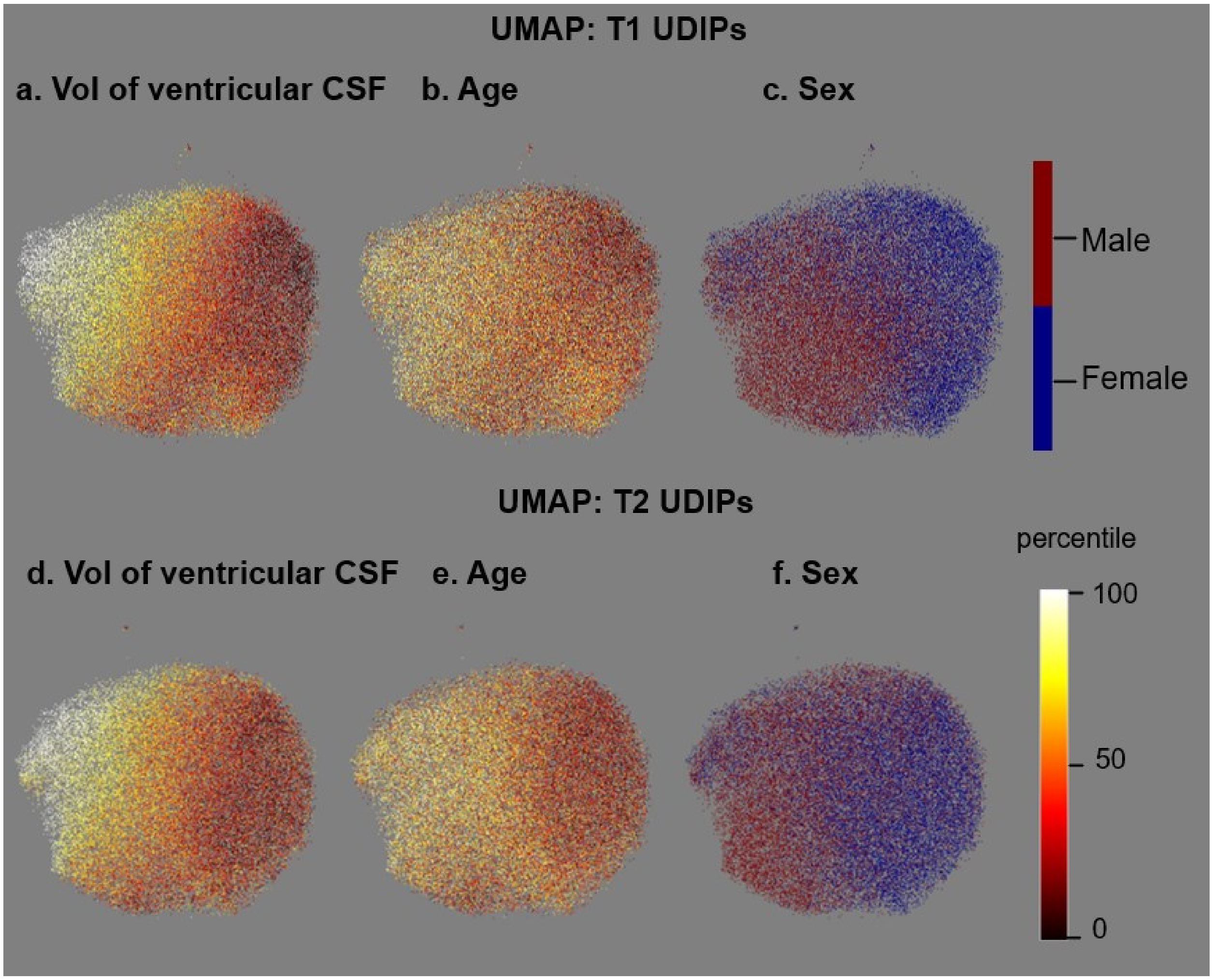

Supplementary Figure 8. Uniform Manifold Approximation and Projection (UMAP) for dimension Reduction of UDIPs.

UDIPs of 37,376 T1 and 36,231 T2-FLAIR MRIs of white British individuals (deep learning test set) are correlated with demographic and brain volume measures as visualized by UMAP. The points are individual participants colored by UKBB provided precomputed features. For a continuous precomputed feature, we convert the value to percentile to make visualization possible. Sex is the only categorical feature.

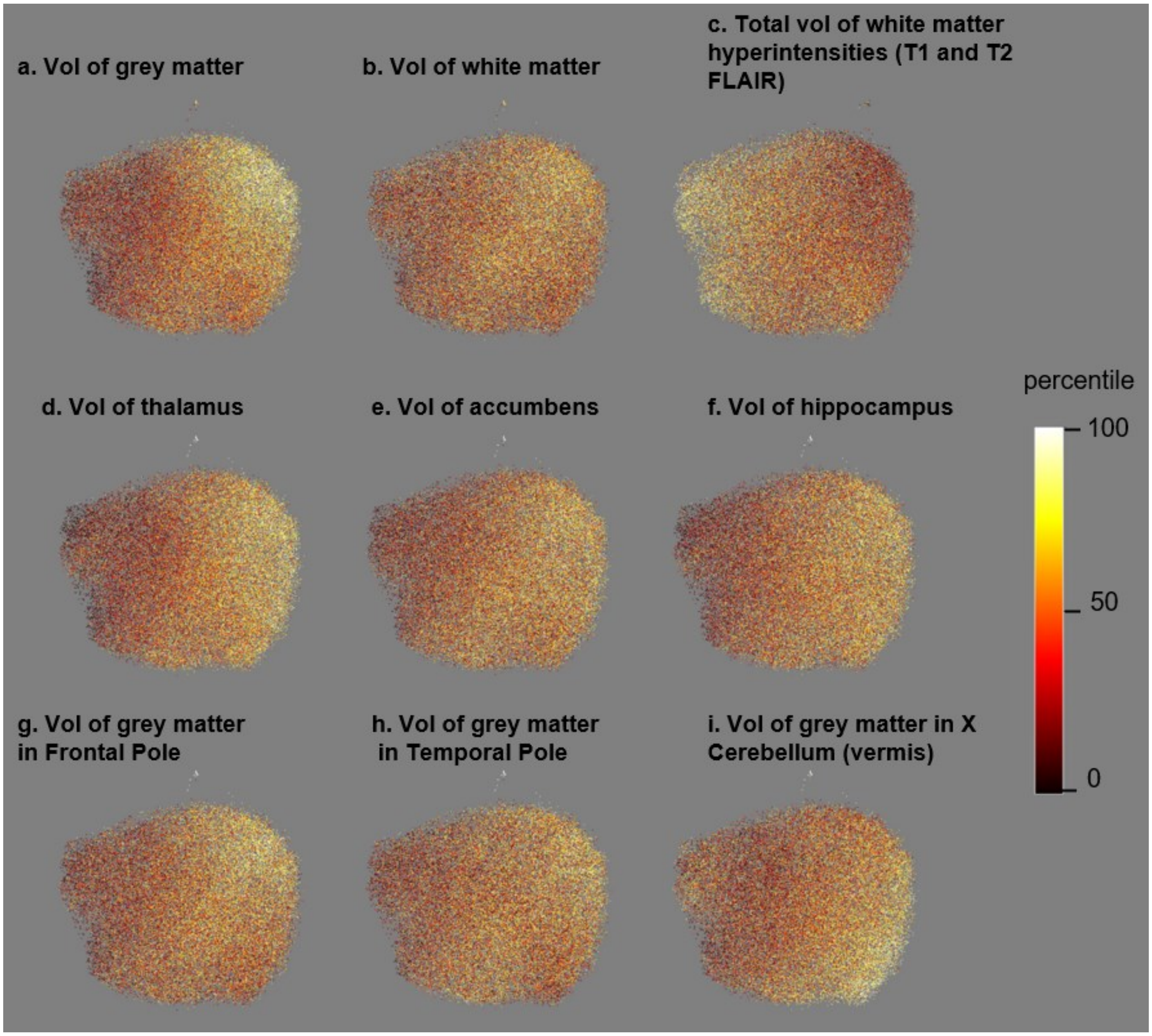

Supplementary Figure 9. UMAP of UDIPs derived from T1. UDIPs of 37,376 T1 MRIs of white British individuals (deep learning test set) are correlated with demographic and brain volume measures as visualized by UMAP. The points are individual participants colored by UKBB provided precomputed features. For a continuous precomputed feature, we convert the value to percentile to make visualization possible. UDIPs of 36,231 T1 MRIs of white British individuals are used for brain volume measure “total volume of white matter hyperintensities” as it is calculated from both T1 and T2. UMAP points colored by volume of ventricular CSF, age and sex.

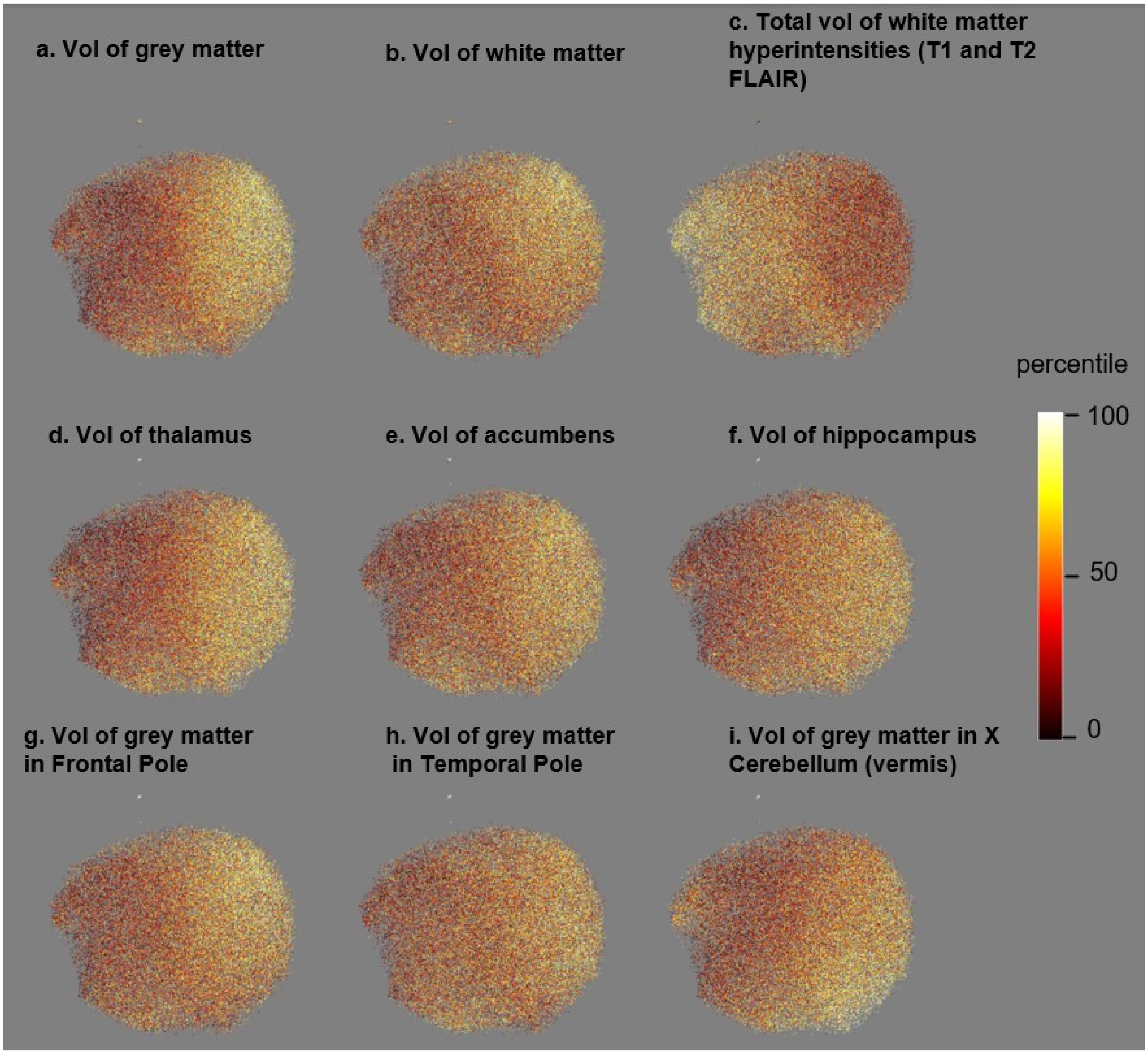

Supplementary Figure 10. UMAP of UDIPs derived from T2-FLAIR. UDIPs of 36,231 T2-FLAIR MRIs of white British individuals (deep learning test set) are correlated with demographic and brain volume measures as visualized by UMAP. The points are individual participants colored by UKBB provided precomputed features. For a continuous precomputed feature, we convert the value to percentile to make visualization possible. UMAP points colored by volume of ventricular CSF, age and sex.

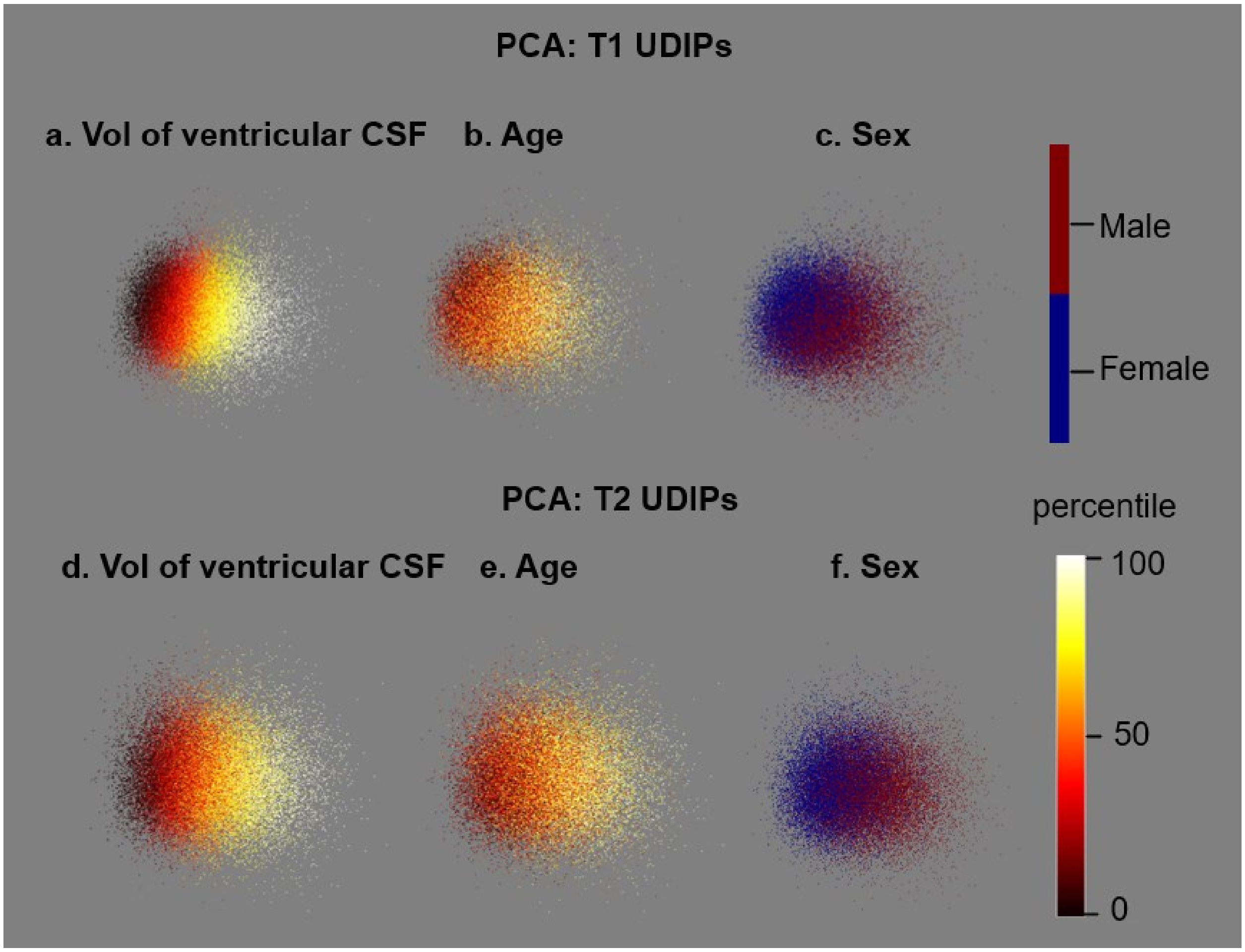

Supplementary Figure 11. Principal component analysis (PCA) dimension reduction for UDIPs.
UDIPs of 37,376 T1 and 36,231 T2-FLAIR MRIs of white British individuals (deep learning test set) are correlated with demographic and brain volume measures as visualized by PCA. The points are individual participants colored by UKBB provided precomputed features. For a continuous precomputed feature, we convert the value to percentile to make visualization possible. Sex is the only categorical feature.

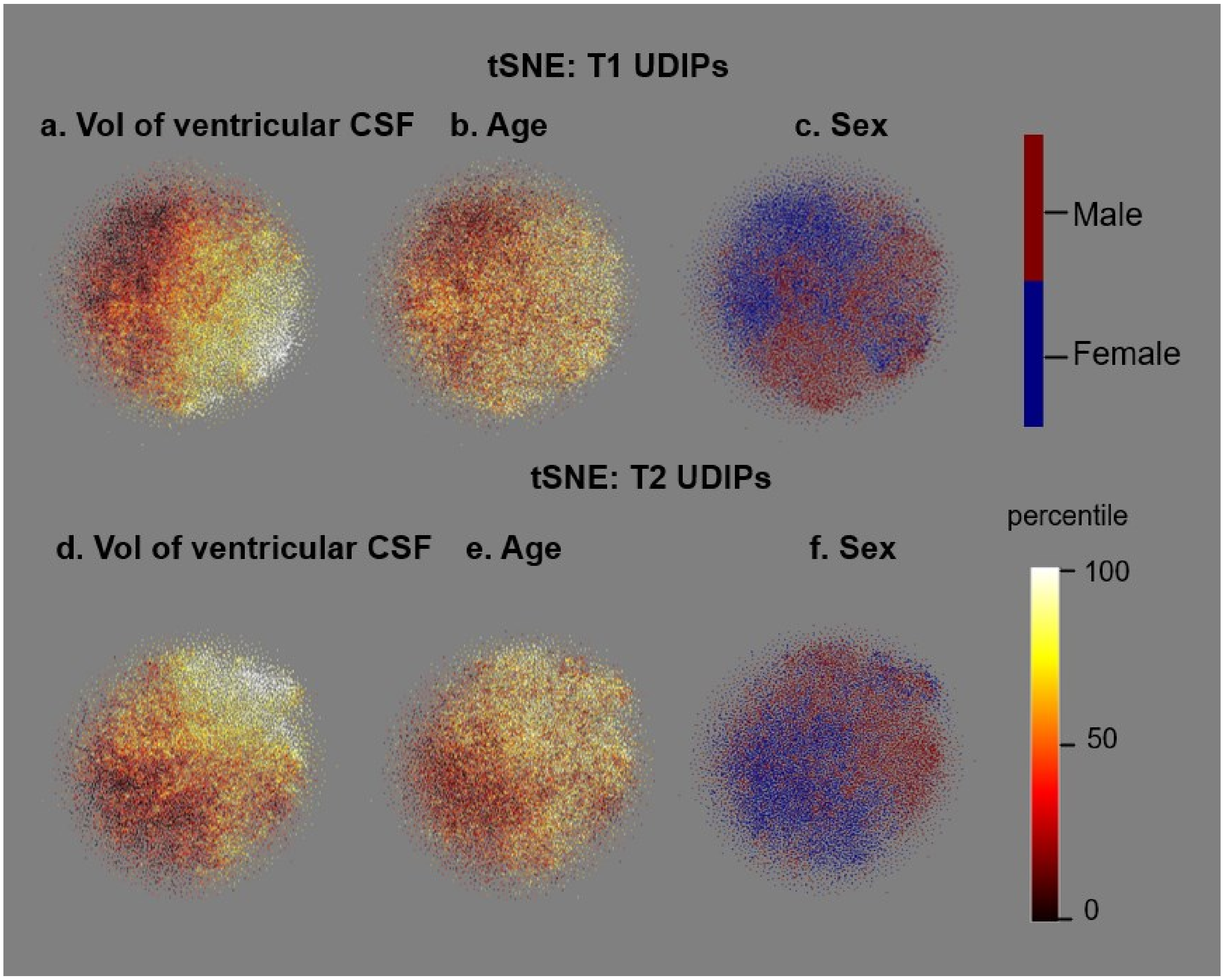

Supplementary Figure 12. t-distributed Stochastic Neighbor Embedding (t-SNE) dimension reduction for UDIPs.

UDIPs of 37,376 T1 and 36,231 T2-FLAIR MRIs of white British individuals (deep learning test set) are correlated with demographic and brain volume measures as visualized by t-SNE. The points are individual participants colored by UKBB provided precomputed features. For a continuous precomputed feature, we convert the value to percentile to make visualization possible. Sex is the only categorical feature.

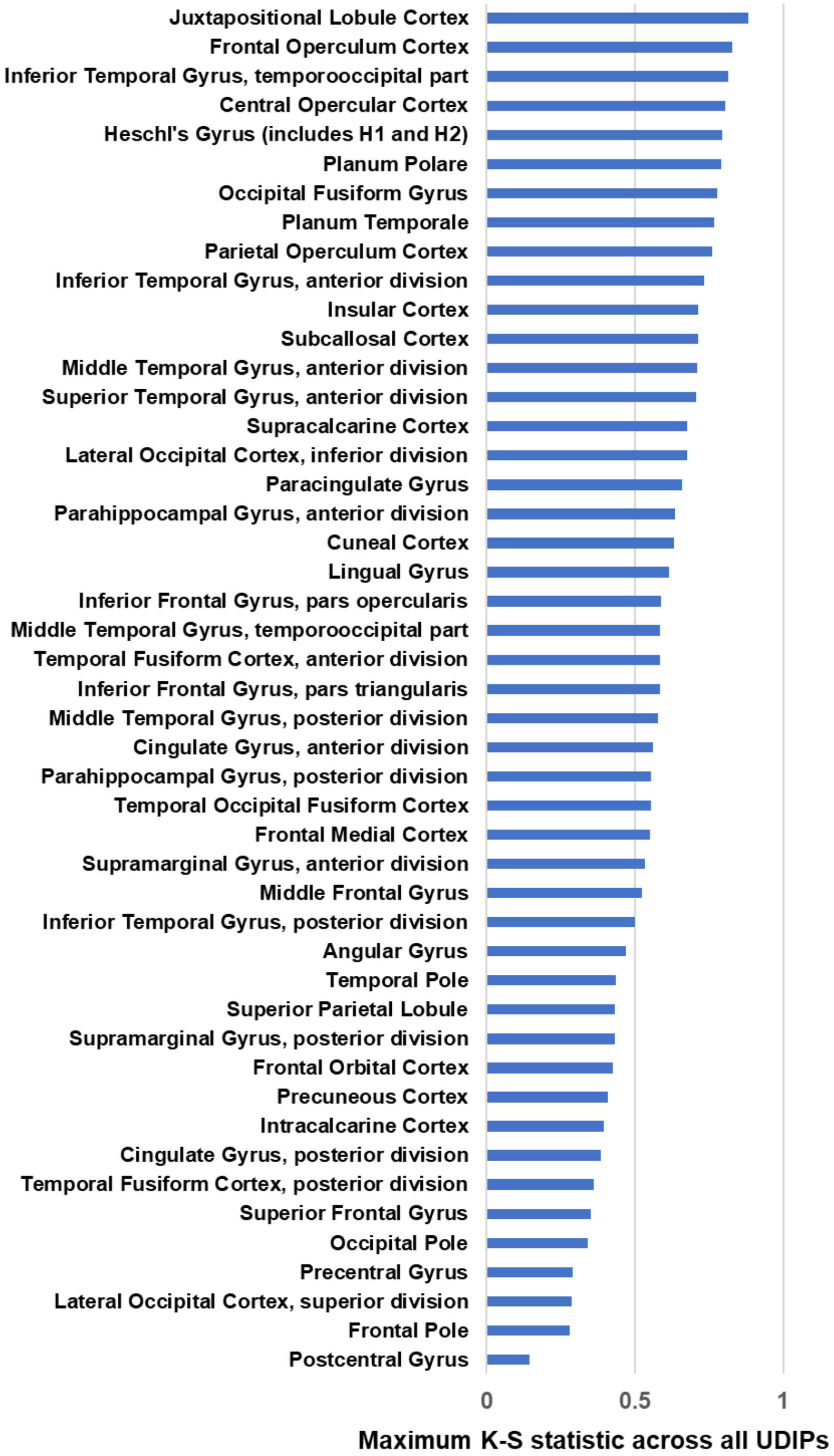

Supplementary Figure 13. Maximum K-S statistic value across all cortical regions in T1. Harvard Oxford cortical atlas (included in FSL) was used to rank voxels in t-map and generate K-S statistic plots.

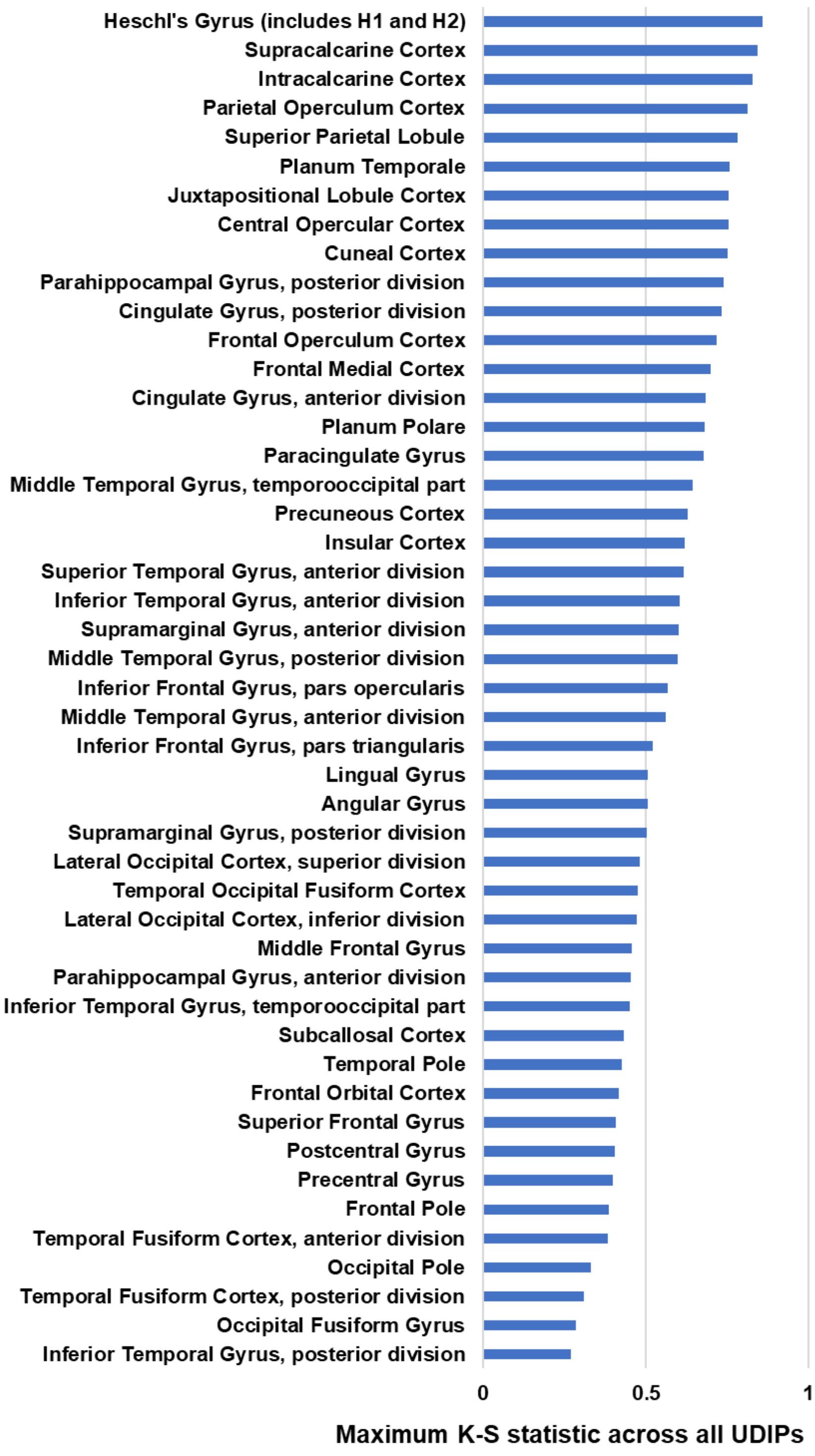

Supplementary Figure 14. Maximum K-S statistic value across all cortical regions in T2-FLAIR.

Harvard Oxford cortical atlas (included in FSL) was used to rank voxels in t-map and generate K-S statistic plots.

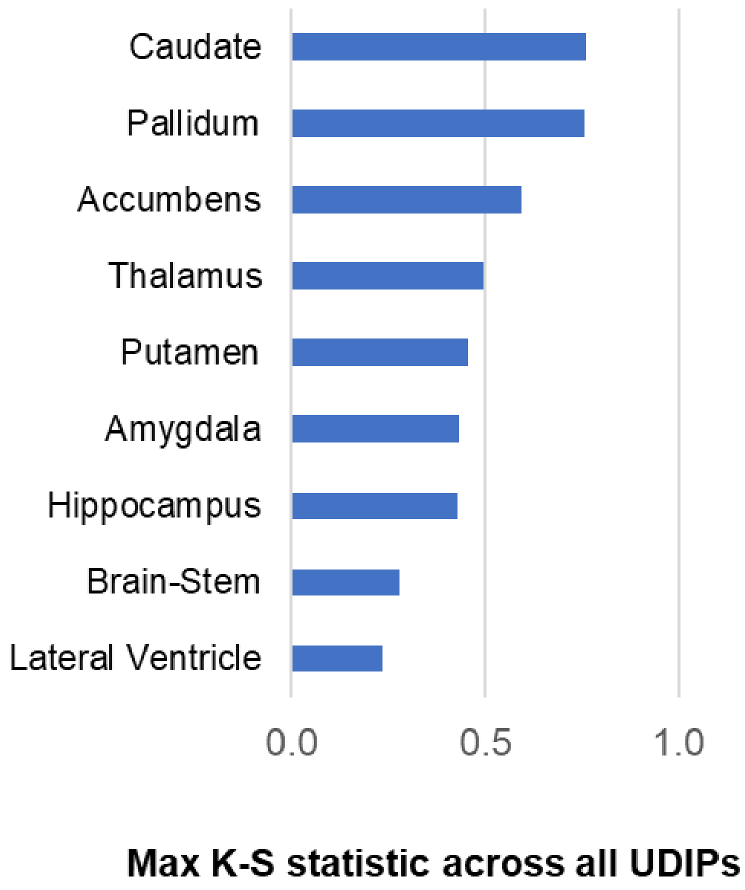

Supplementary Figure 15. Maximum K-S statistic value across all subcortical regions in T1.

Harvard Oxford subcortical atlas (included in FSL) was used to rank voxels in t-map and generate K-S statistic plots. Caudate, pallidum, accumbens, thalamus, amygdala, hippocampus and putamen have maximum K-S statistic value of >0.5 and are represented well.

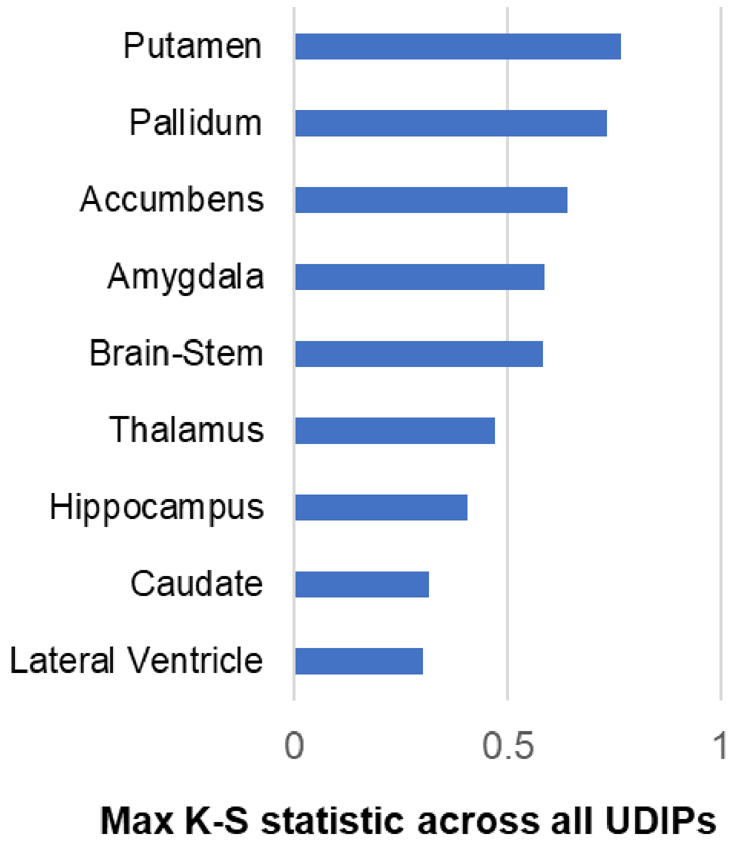

Supplementary Figure 16. Maximum K-S statistic value across all subcortical regions in T2.
Harvard Oxford subcortical atlas (included in FSL) was used to rank voxels in t-map and generate K-S statistic plots. Caudate, pallidum, accumbens, thalamus, amygdala, hippocampus and putamen have maximum K-S statistic value of >0.5 and are represented well.

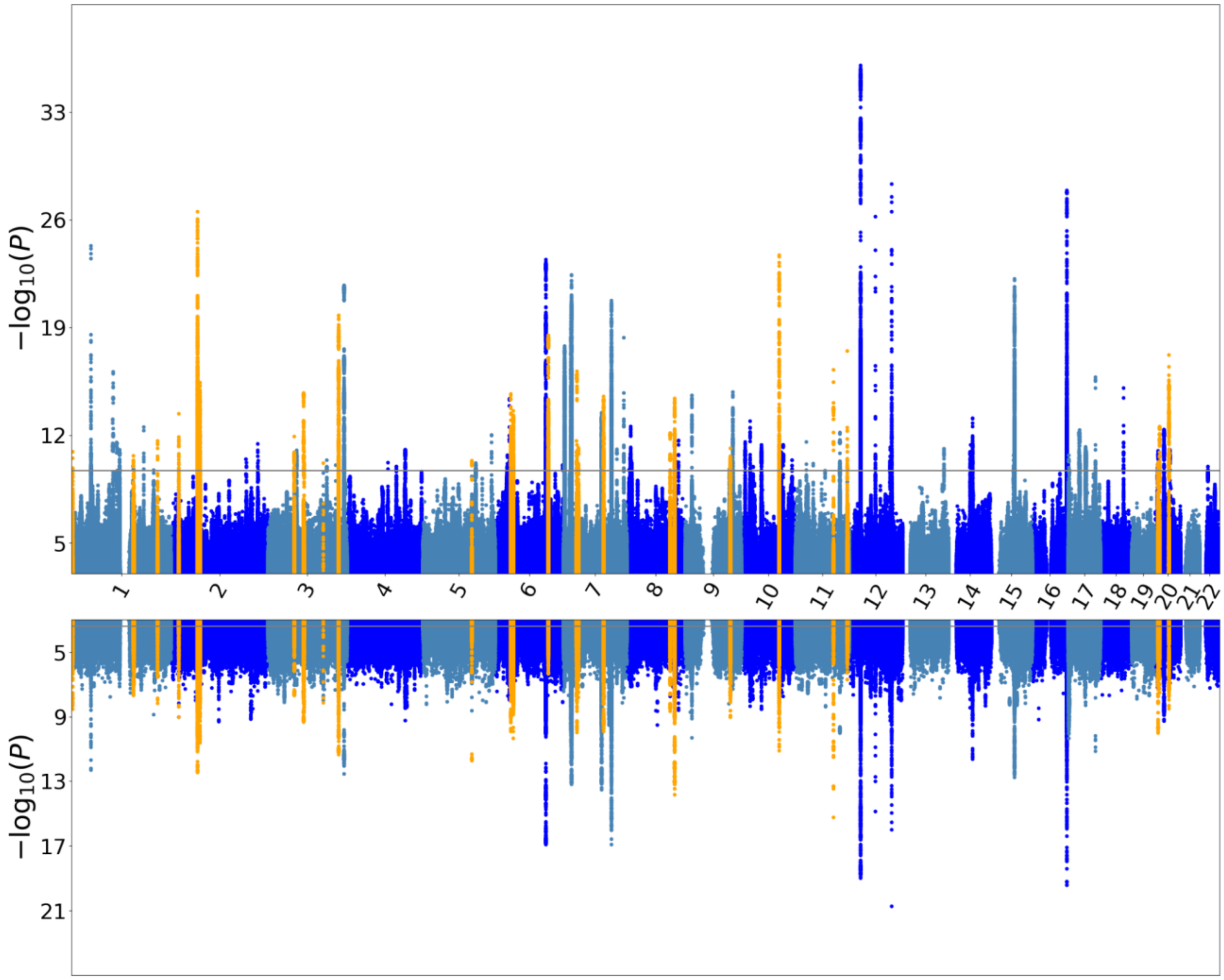

Supplementary Figure 17. Miami plot of GWAS results in discovery and replication cohorts.

Aggregated Miami plot of all 256 single UDIP GWASs is shown. Top panel is the Manhattan plot from the GWAS of the discovery cohort, the black line marks the genome-wide significance level at 5e-8/256 =1.953e-10. Bottom panel is the Manhattan plot from the GWAS of the replication cohort, the black line marks the significance level at 0.05/126=0.000396, where 126 is the number of lead SNPs in the discovery cohort. 26 loci not reported in earlier UK Biobank T1 and T2 IDP GWAS are colored yellow.

a.

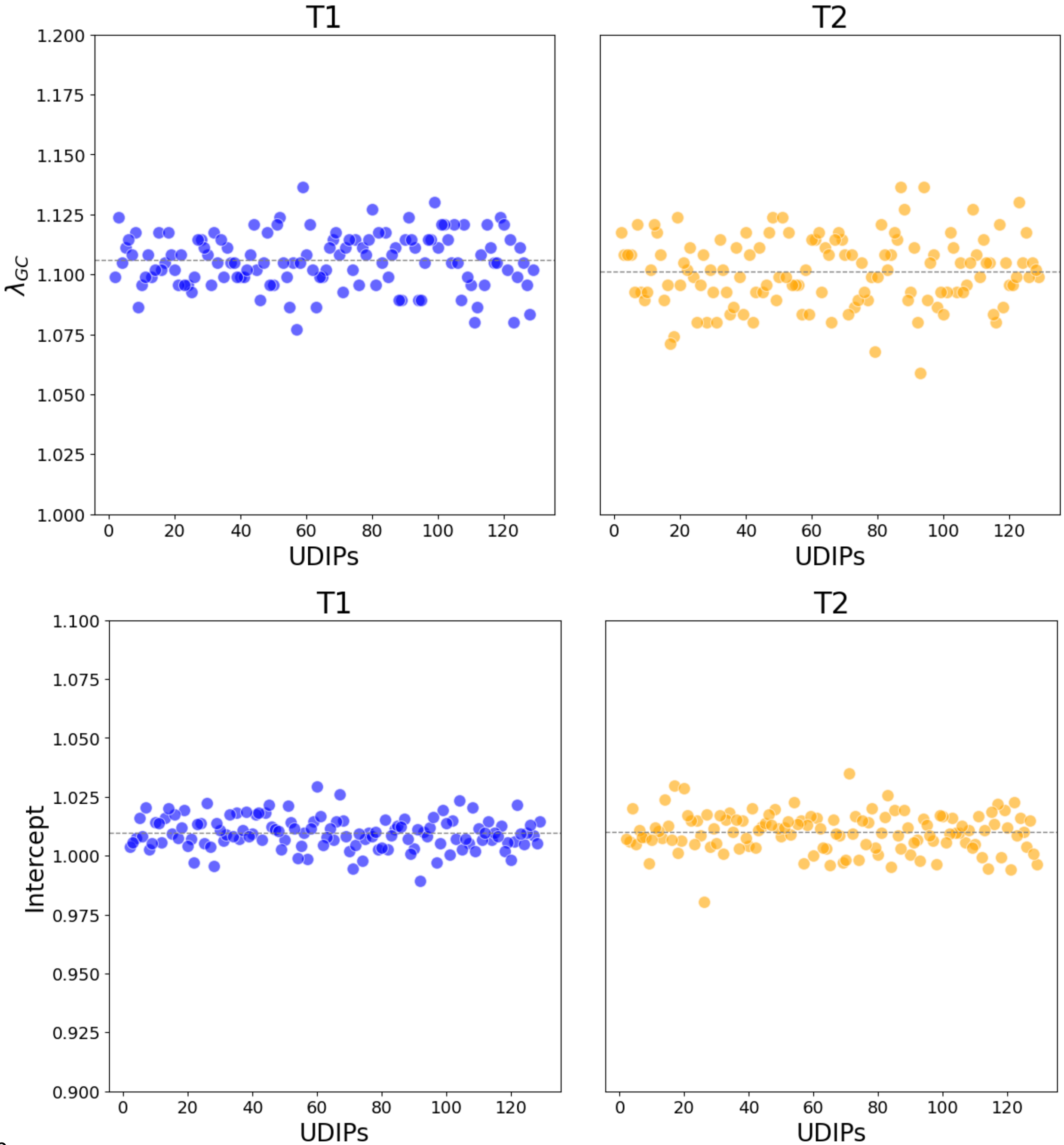

b.

Supplementary Figure 18. Distribution of λ_{GC} and LDSC Intercepts Across Different Dimensions

a. Genomic inflation factor (λ_{GC}): No genetic information was used while training the deep learning models which resulted in well well-controlled genomic inflation factor with mean λ_{GC} for T1 (1.106 ± 0.011) and T2 (1.101 ± 0.014) indicated through dashed lines **b.** Mean LDSC intercept values for T1 (1.009 ± 0.008) and T2 (1.009 ± 0.007) indicated through dashed lines for both modalities are close to 1.

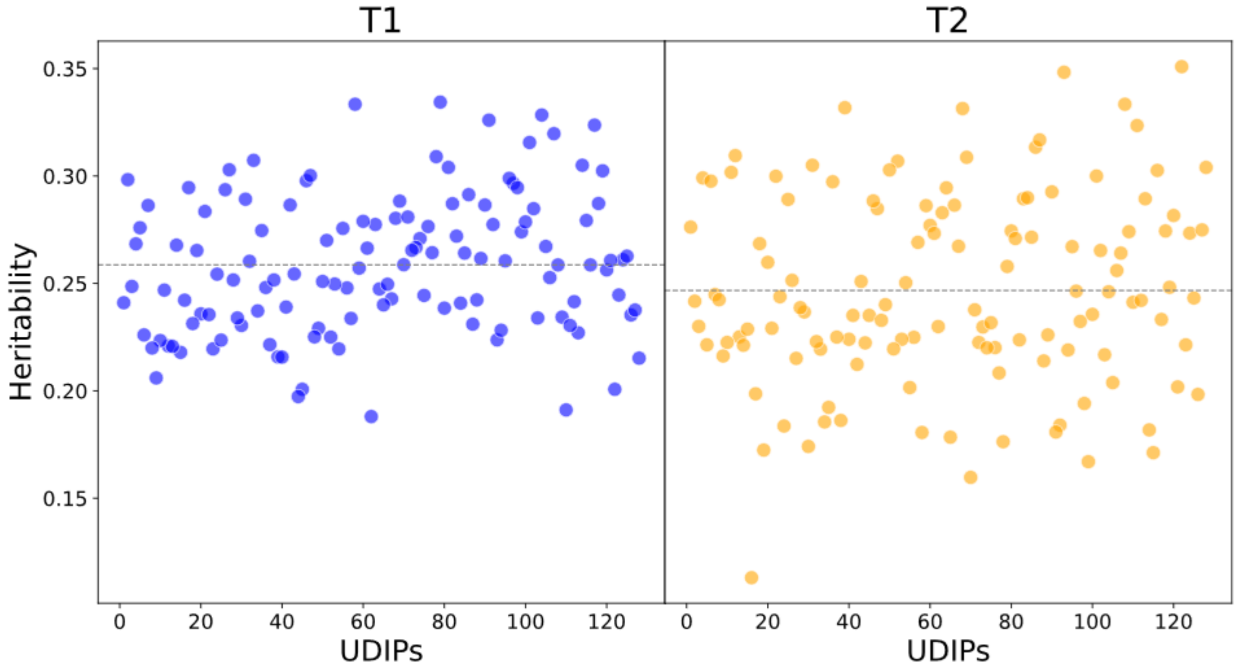

Supplementary Figure 19. Heritabilities of UDIPs.
Heritability is calculated using LDSC with mean LDSC for T1 (0.258±0.032) and T2 (0.247±0.045) indicated through dashed lines.

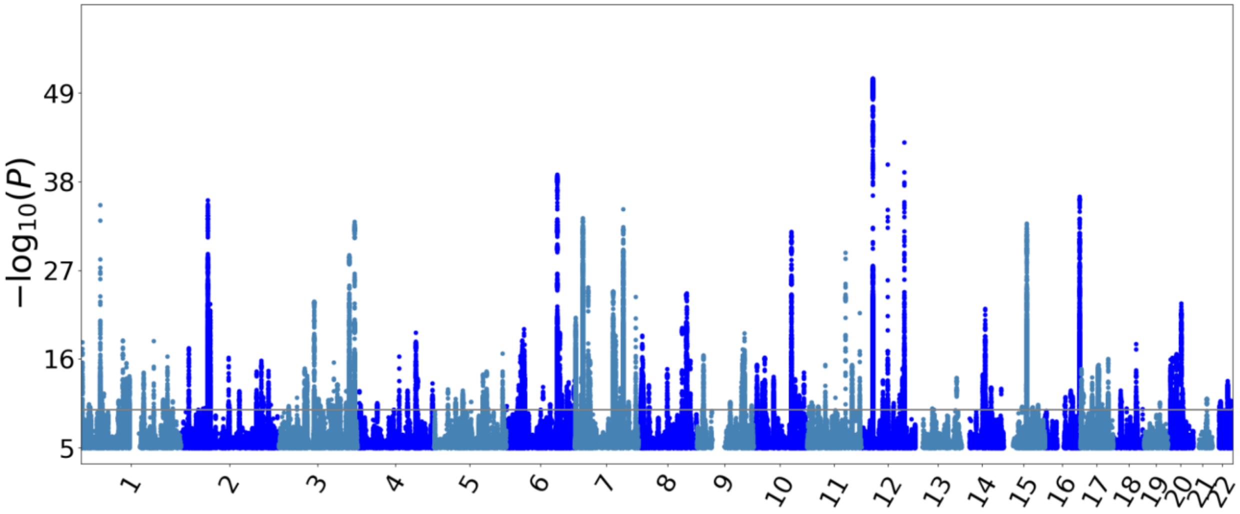

Supplementary Figure 20. Meta-analysis Manhattan plot.
Sample size weighted fixed-effect meta-analysis of the discovery and the replication GWAS summary statistics identified 95,061 significant ($P<5x10^{-8}/256$) SNP-UDIP pairs involving 19,617 SNPs clustered into 199 loci.

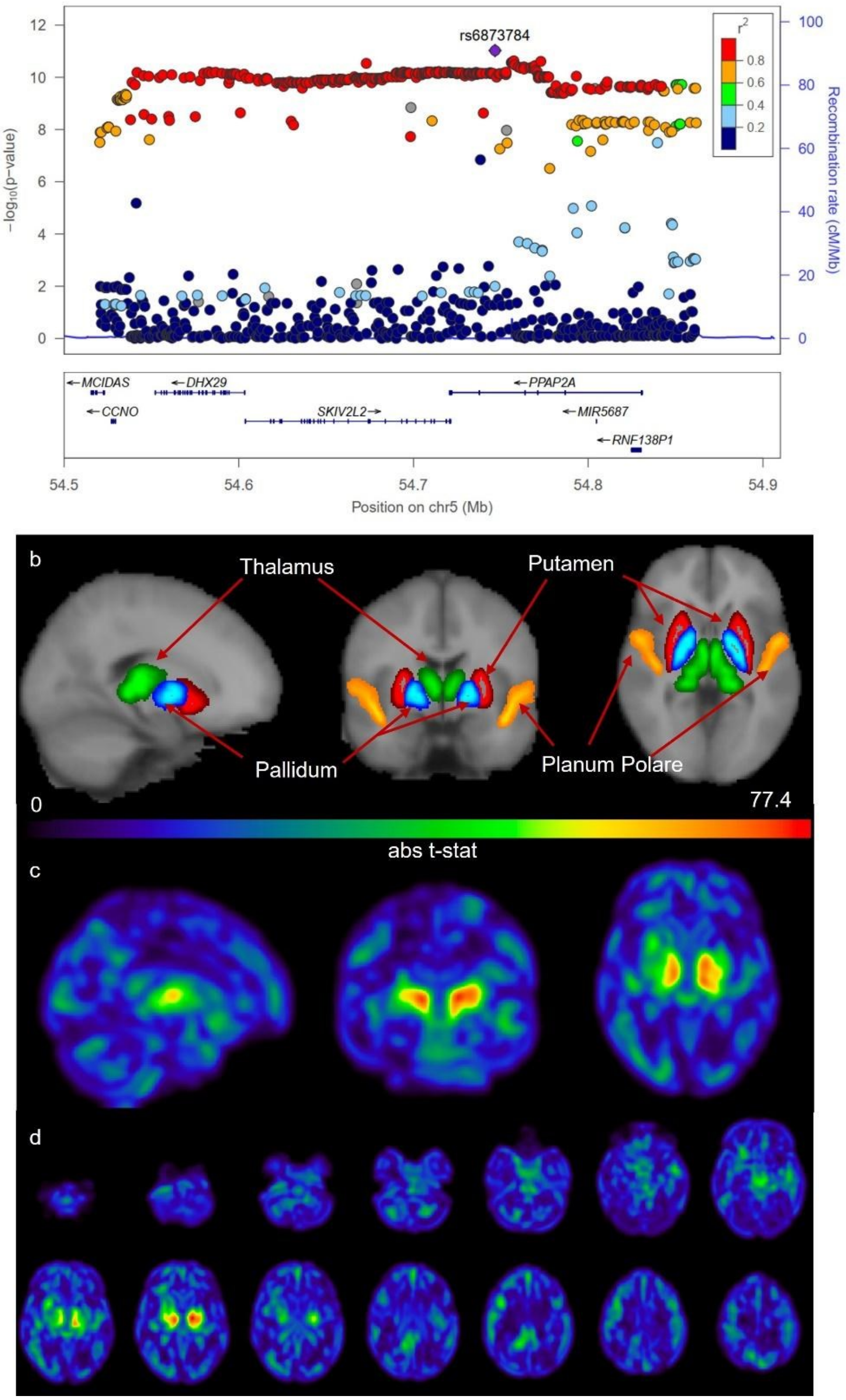

Supplementary Figure 21. Lead UDIP T2:93 identifies a new locus on chromosome 5 that was not previously associated with brain-related traits.

a) Regional plot for lead SNP rs6873784 is identified by meta-analysis. b) Harvard-Oxford structural atlas is utilized for region annotation. c) t-map shows pallidum, putamen, and thalamus as the most prominent subcortical structures, whereas planum polare is the most prominent cortical structure. d) Lightbox view (axial) displays t-map slices across the entire brain.

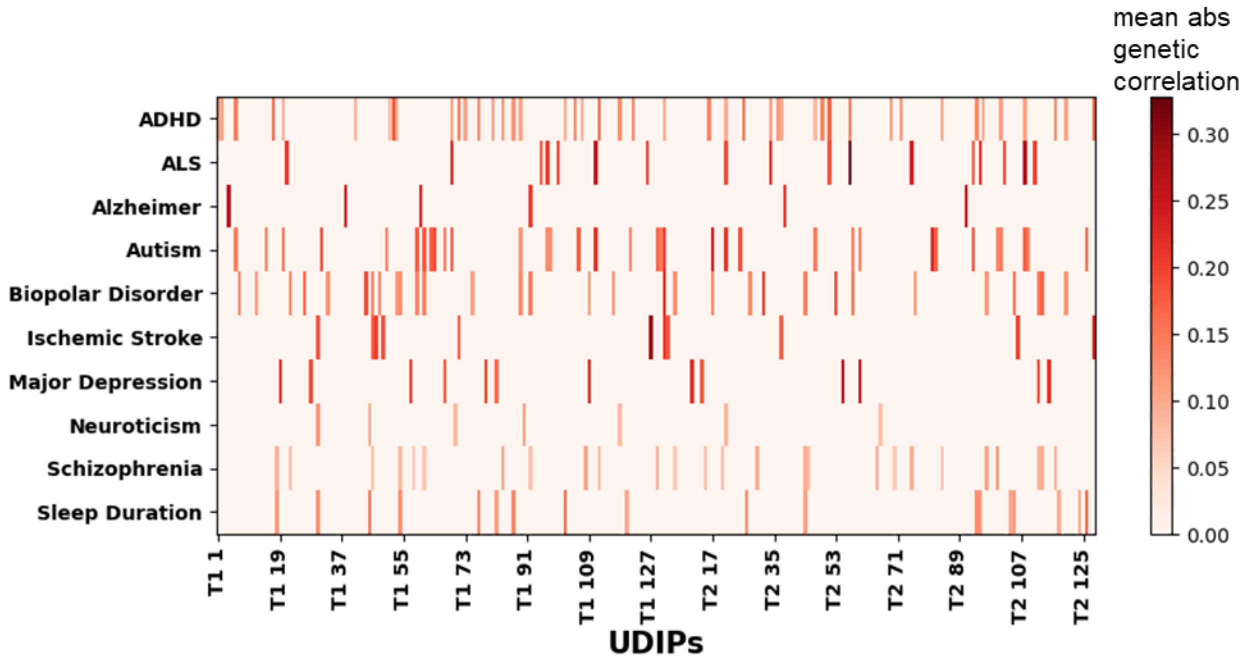

Supplementary Figure 22. Mean absolute genetic correlation of common brain-related diseases with UDIPs using meta-analysis results (Discovery and replication cohort).
Mean absolute genetic correlation having $p < 0.05$ using meta-analysis results.
ADHD: Attention-deficit/hyperactivity disorder, ALS: Amyotrophic lateral sclerosis

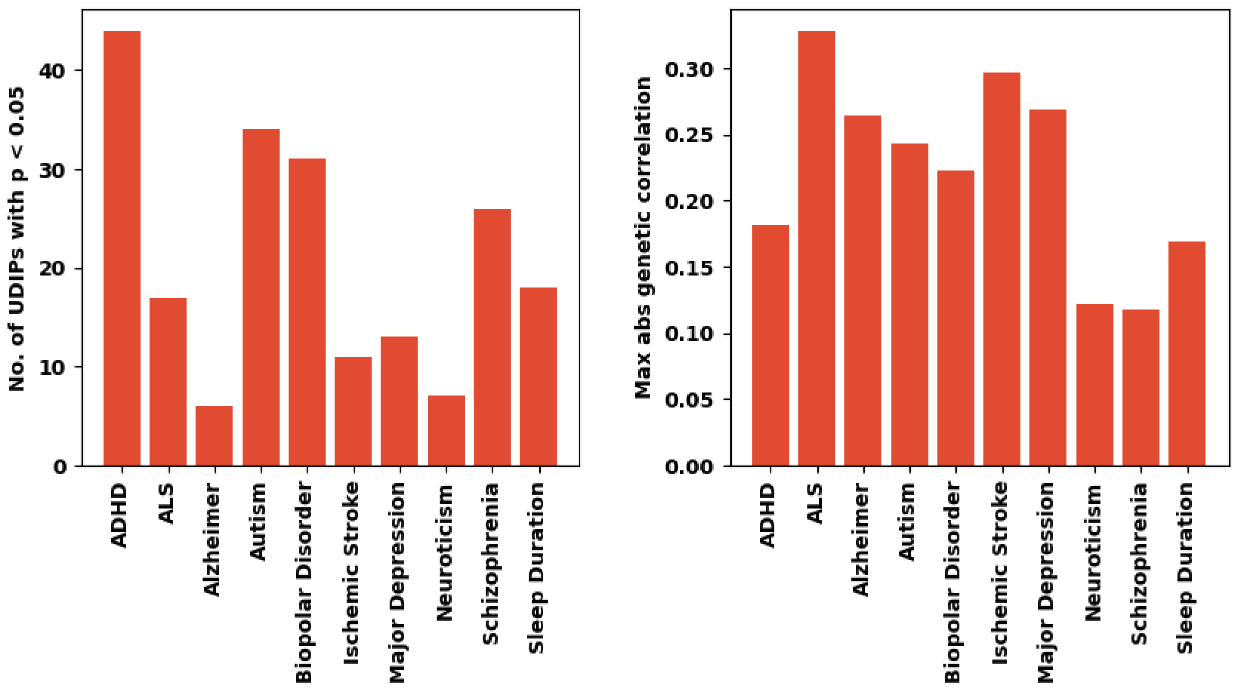

Supplementary Figure 23.

a. Number of UDIPs with p-value <0.05 for genetic correlation results for various diseases b. Max absolute genetic correlation for various brain-related conditions.

ADHD: Attention-deficit/hyperactivity disorder, ALS: Amyotrophic lateral sclerosis

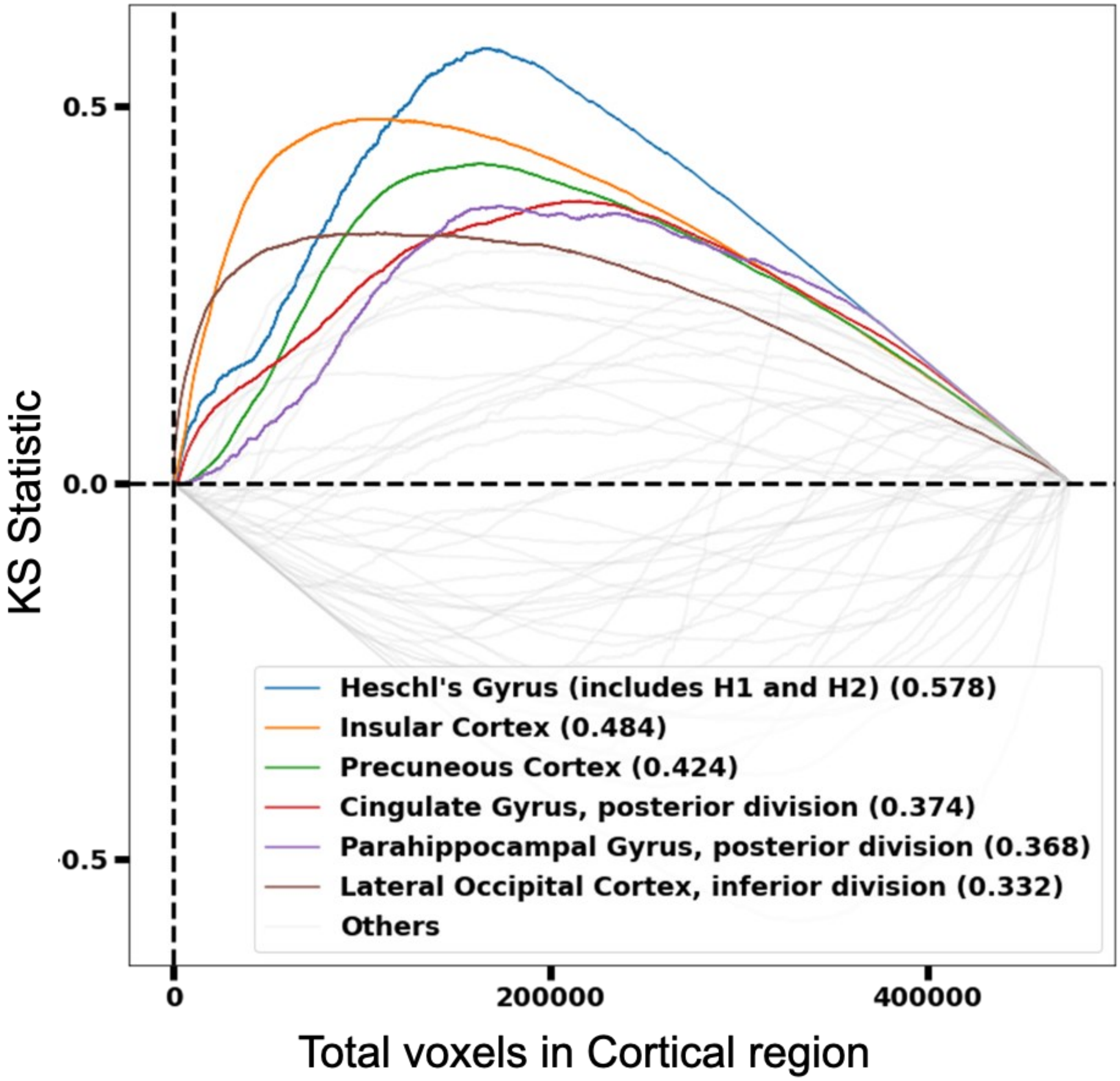

Supplementary Figure 24. K-S statistics plot for cortical region t-map for UDIP T2:67. Harvard-Oxford cortical atlas was used to select regions of t-map generated through PerDI for UDIP T2:67. Voxels in the cortical atlas were ranked and K-S statistic plot was generated.

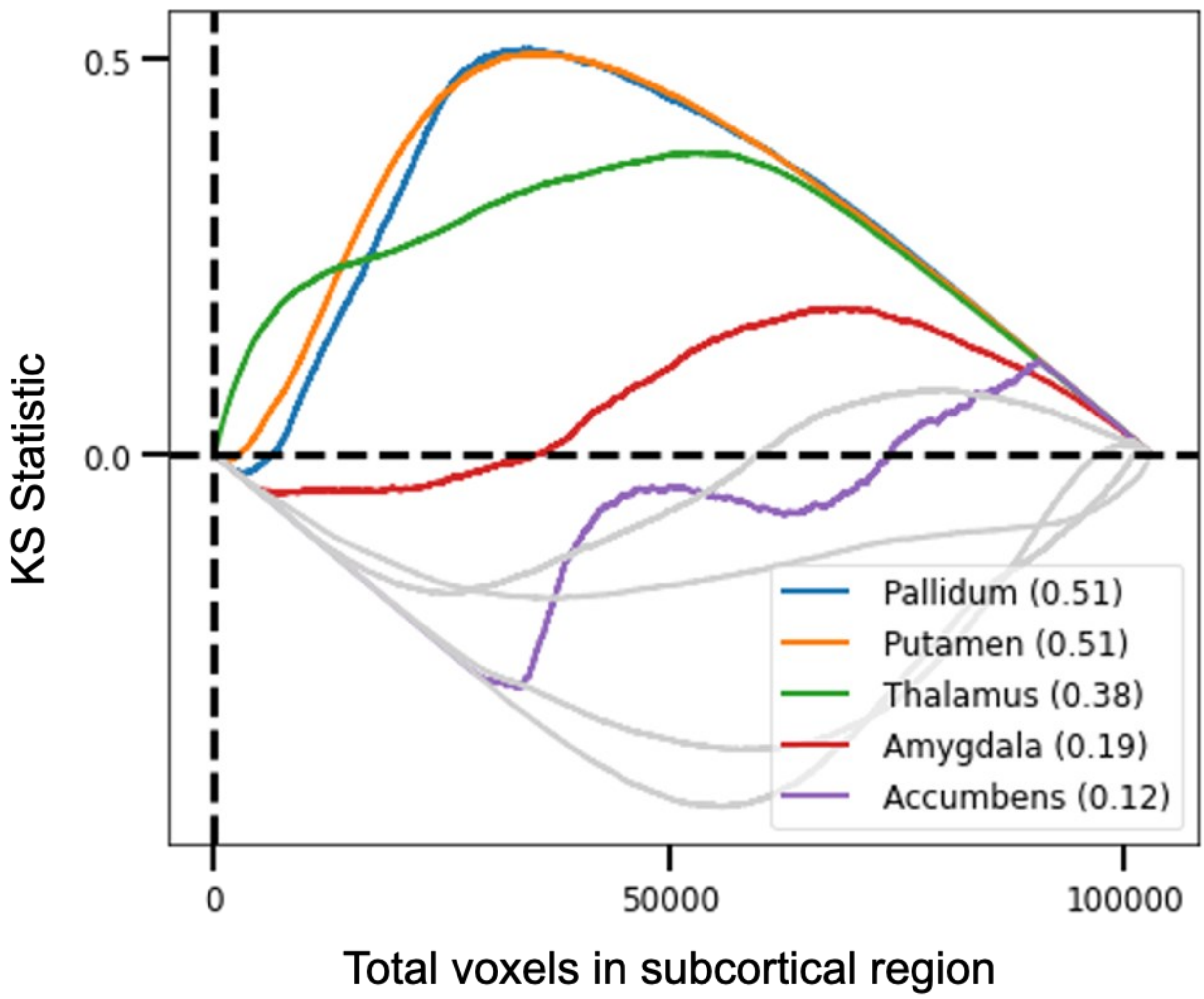

Supplementary Figure 25. K-S statistics plot for subcortical region t-map for UDIP T2:67. Harvard-Oxford subcortical atlas was used to select regions of t-map generated through PerDI for UDIP T2:67. Voxels in the subcortical atlas were ranked and K-S statistic plot was generated.

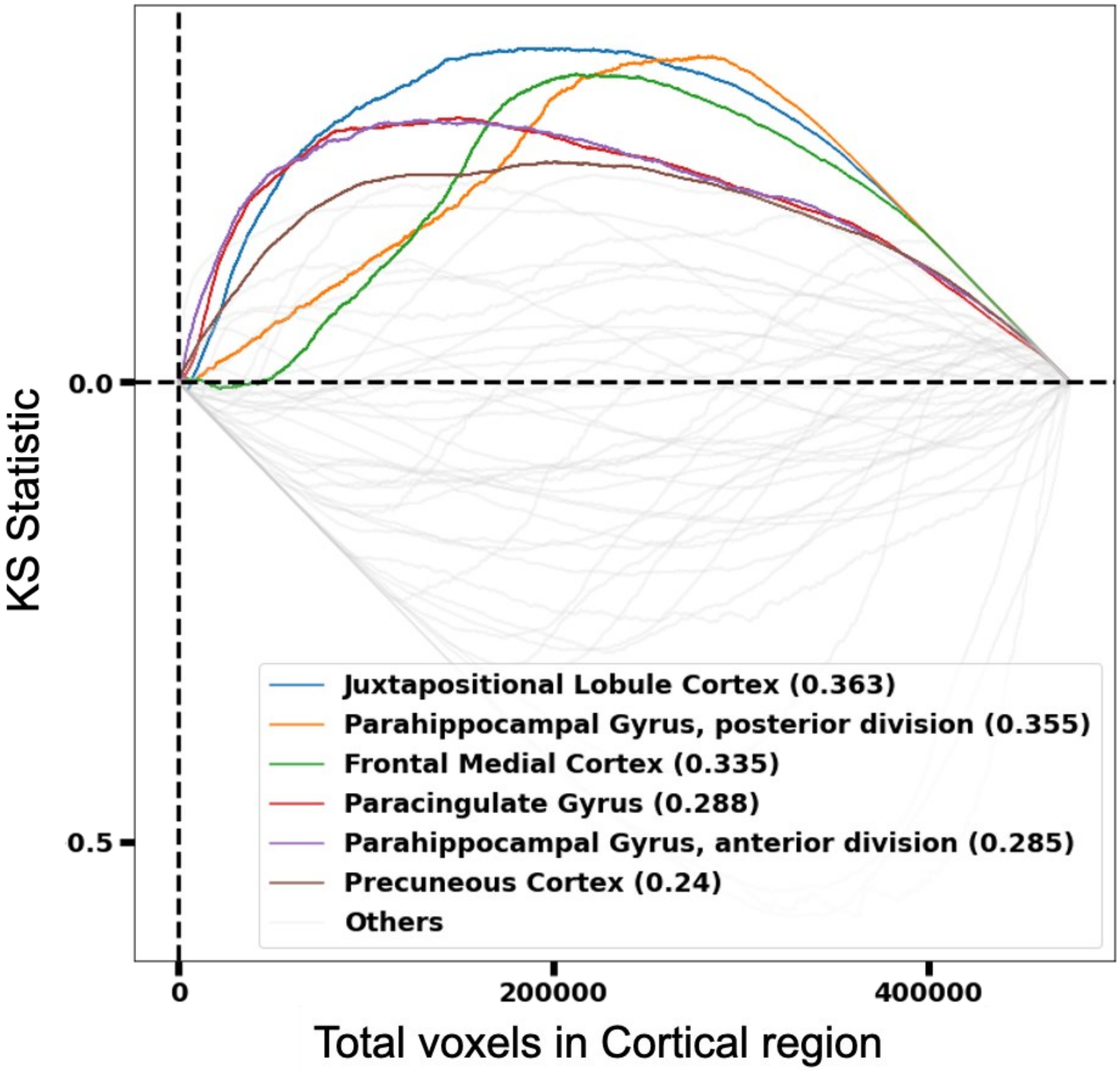

Supplementary Figure 26. K-S statistics plot for cortical region t-map for UDIP T2:14. Harvard-Oxford cortical atlas was used to select regions of t-map generated through PerDI for UDIP T2:14. Voxels in the cortical atlas were ranked and K-S statistic plot was generated.

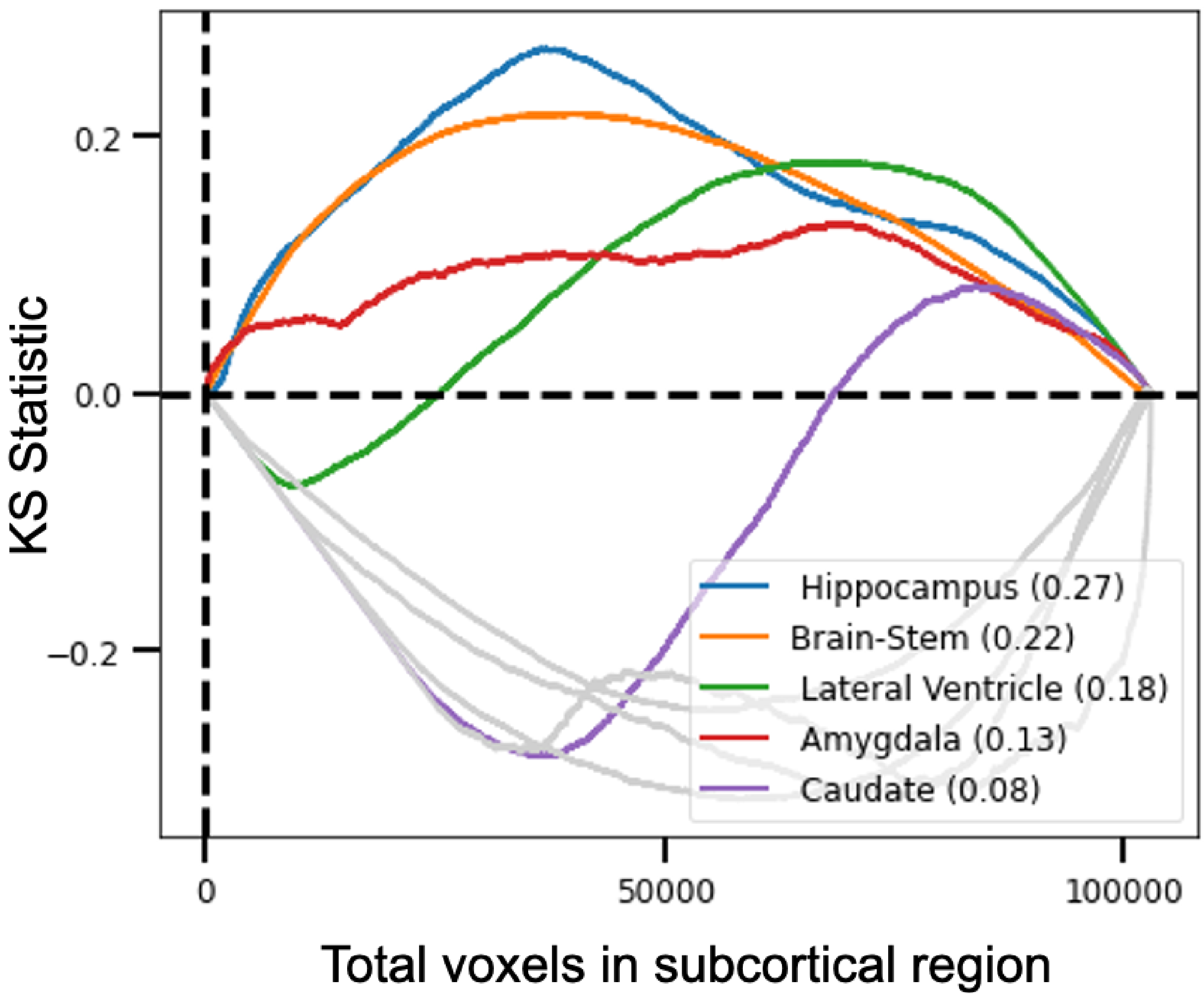

Supplementary Figure 27. K-S statistics plot for subcortical region t-map for UDIP T2:14. Harvard-Oxford subcortical atlas was used to select regions of t-map generated through PerDI for UDIP T2:14. Voxels in the subcortical atlas were ranked and K-S statistic plot was generated.

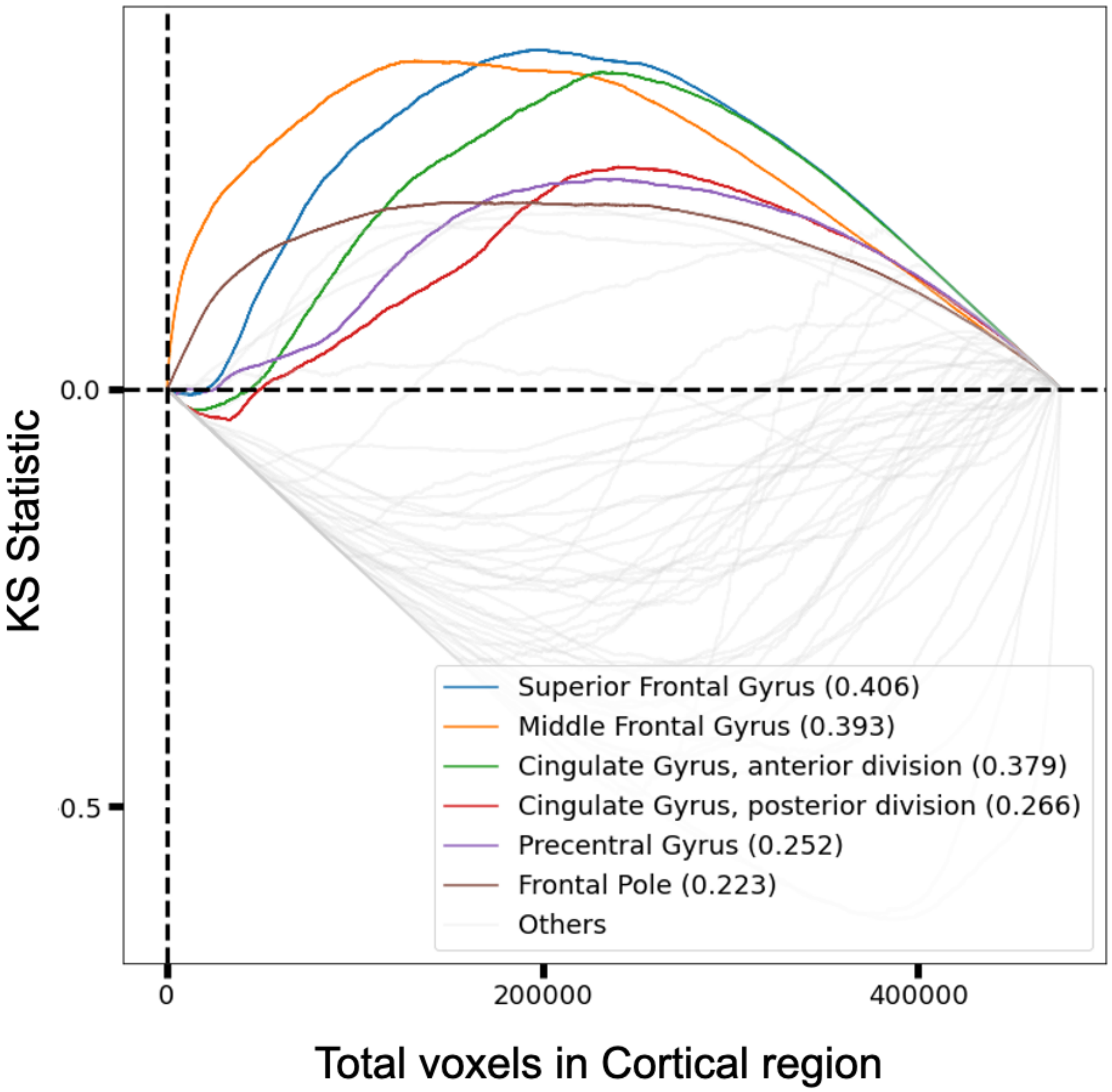

Supplementary Figure 28. K-S statistics plot for cortical region t-map for UDIP T2:64. Harvard-Oxford cortical atlas was used to select regions of t-map generated through PerDI for UDIP T2:64. Voxels in the cortical atlas were ranked and K-S statistic plot was generated.

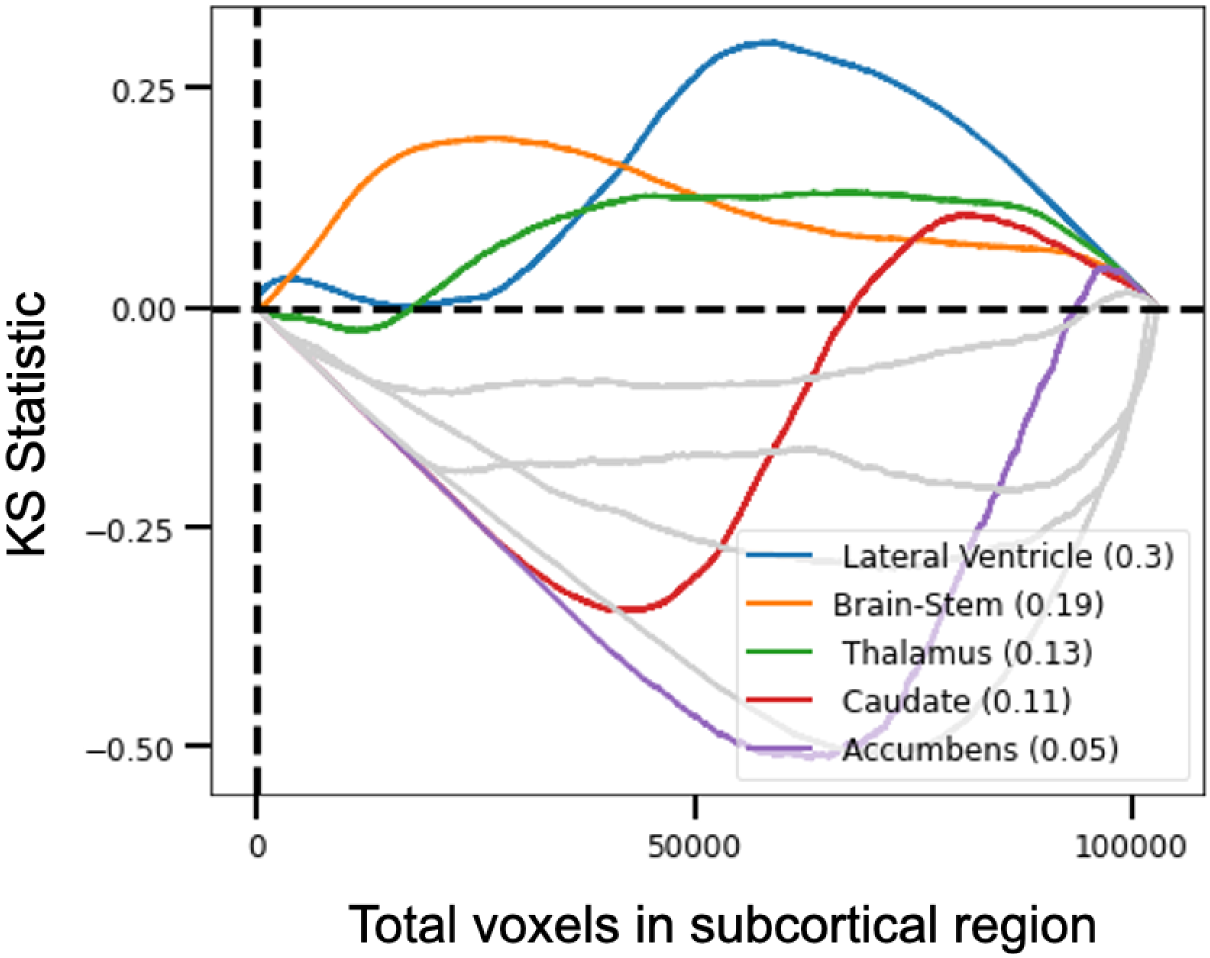

Supplementary Figure 29. K-S statistics plot for subcortical region t-map for UDIP T2:64. Harvard-Oxford subcortical atlas was used to select regions of t-map generated through PerDI for UDIP T2:64. Voxels in the subcortical atlas were ranked and K-S statistic plot was generated.

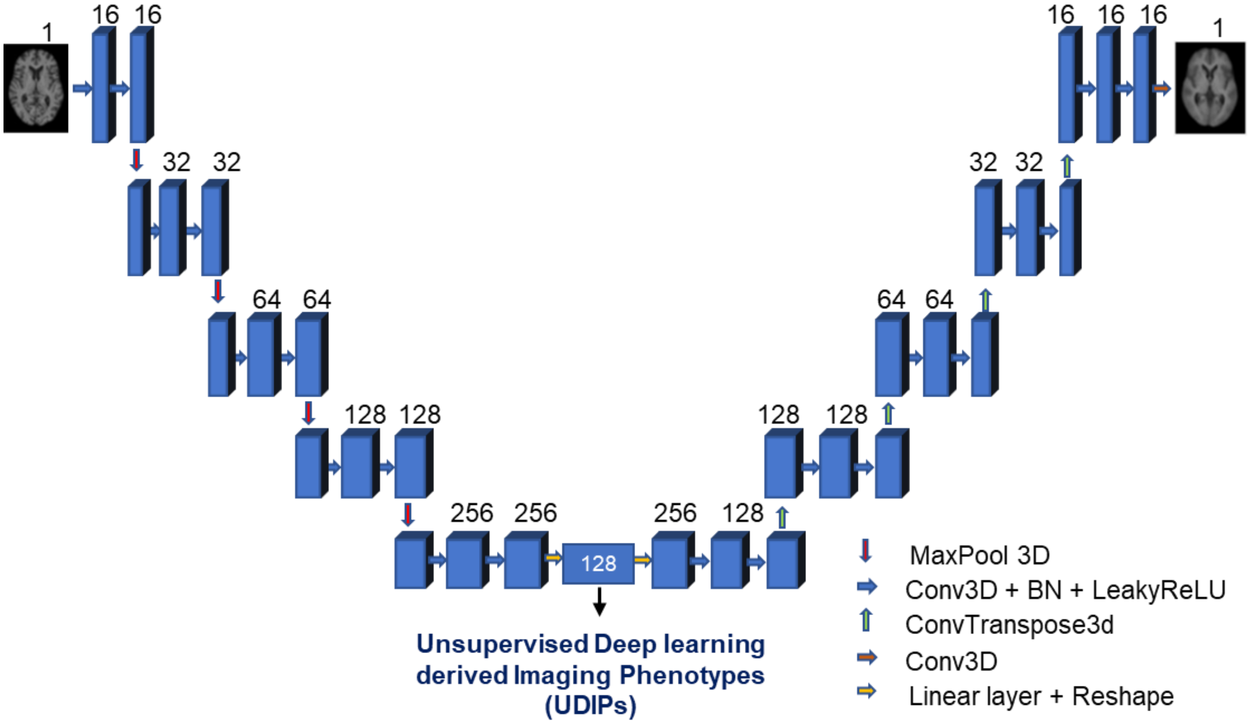

Supplementary Figure 30. 3D convolutional autoencoder architecture.
3D convolutional autoencoder comprises four blocks of encoder and 4 blocks of decoder using 3D convolutional layers. 128-dimensional latent space is used as imaging phenotypes for GWAS.

Supplementary References

1. Watanabe, K., Taskesen, E., van Bochoven, A. & Posthuma, D. Functional mapping and annotation of genetic associations with FUMA. *Nat. Commun.* **8**, 1826 (2017).
