# Supplementary material for: Unsupervised deep representation learning enables phenotype discovery for genetic association studies of brain imaging
Source: Commun Biol. 2024 Apr 5;7:414. doi: 10.1038/s42003-024-06096-7 (PMC10997628; doi:10.1038/s42003-024-06096-7)
Supplement: Supplementary file 2 — Description of Supplementary Materials file [file 42003_2024_6096_MOESM2_ESM.docx]

**Description of Additional Supplementary Files**

**File name:** Supplementary Data 1

**Description:** Sample sizes of all data sets used in this study.

**File name:** Supplementary Data 2

**Description:** Loci table for discovery-replication cohort.

**File name:** Supplementary Data 3

**Description:** Regression R^2^ values for UK Biobank provided precomputed phenotypes obtained using standard neurology software packages.

**File name:** Supplementary Data 4

**Description:** Description of outliers in UMAP of T1 UDIP and T2 FLAIR UDIP.

**File name:** Supplementary Data 5

**Description:** Supplementary Data 5. a: Absolute Pearson Correlation value calculated between 128 T1 derived UDIPs and traditional imaging derived phenotypes obtained from UKBiobank. Supplementary Data 5. b: Absolute Pearson Correlation value calculated between 128 T2 derived UDIPs and traditional imaging derived phenotypes obtained from UKBiobank.

**File name:** Supplementary Data 6

**Description:** Description of Pearson correlation between UMAP components of T1 UDIPs and T2 FLAIR UDIPs. UMAP reduces 128 dimensional T1 UDIPs and 128 dimensional T2 UDIPs into 2 components. The 2 components of T1 and T2 are correlated with each other.

**File name:** Supplementary Data 7

**Description:** Effect of change of latent space dimension. a) Effect of latent space dimension (number of UDIPs) on reconstruction MSE and variance explained (Canonical Correlation Analysis (CCA)) b) Effect of latent space dimension (number of UDIPs) on number of loci identified through GWAS of 22,800 T1 MRI (discovery set)

**File name:** Supplementary Data 8

**Description:** K-S statistic values for T1 and T2-FLAIR

Perturbation based approach provides K-S statistic values for T1 and T2-FLAIR UDIPs using Harvard-Oxford cortical and subcortical atlas. Higher K-S statistic values for a specific UDIP suggest more representation of the region by the UDIP. Each modality has a separate sheet for cortical and subcortical regions

**File name:** Supplementary Data 9

**Description:** GWAS catalog results obtained from querying discovery cohort GWAS hits. Refer to Methods: Querying GWAS Catalog for more information. Refer to Supplementary Data 2 for details on individual locus.

**File name:** Supplementary Data 10

**Description:** Genes identified by FUMA for T2-Flair. Based on GWAS summary statistics, FUMA mapped genes based on the position of Lead SNP, Independent Significant SNP.

**File name:** Supplementary Data 11

**Description:** Gene-set enrichment result by FUMA for T2-Flair. Based on GWAS summary statistics, FUMA mapped genes based on the position of Lead SNP, Independent Significant SNP. With all the unique genes associated with 128 UDIP, gene-set enrichment analysis was performed in FUMA

**File name:** Supplementary Data 12

**Description:** Genes identified by FUMA for T1. Based on GWAS summary statistics, FUMA mapped genes based on position of Lead SNP, Independent Significant SNP.

**File name:** Supplementary Data 13

**Description:** Gene-set enrichment result by FUMA for T1. Based on GWAS summary statistics, FUMA mapped genes based on the position of Lead SNP, Independent Significant SNP. With all the unique genes associated with 128 ENDOs, gene-set enrichment analysis was performed in FUMA.

**File name:** Supplementary Data 14

**Description:** Loci table for meta-analysis cohort.

**File name:** Supplementary Data 15

**Description:** Source code and data to create Fig 3.d.
